# Cooperative R&D contracts for power batteries under R&D outcome uncertainty and subsidies

**DOI:** 10.1371/journal.pone.0362349

**Published:** 2026-11-11

**Authors:** Jinian Liu, Haiyan Chen, Yang Cao

**Affiliations:** 1 School of Law and Business, Hubei Business College, Wuhan, Hubei, China; 2 School of Finance and Accounting, Anhui Finance & Trade Vocational College, Hefei, Anhui, China; Middle Tennessee State University Jennings A Jones College of Business, UNITED STATES OF AMERICA

## Abstract

This paper examines a supply chain comprising a single new energy vehicle manufacturer (NEVM) and a single power battery supplier (PBS), considering government subsidies and R&D outcome uncertainty. An exponential function is employed to characterize R&D outcome uncertainty. The impacts of R&D subsidies and R&D capability on R&D investment, supply chain members, and their expected profits are examined. A comparative analysis of cost-sharing (CS) and revenue-sharing (RS) contracts is conducted. The findings reveal that: (1) R&D investment exhibits an inverted U-shaped relationship with R&D capability and a positive relationship with R&D subsidies. (2) Under an RS contract, the expected profits of the supply chain and its members increase with higher R&D subsidies and enhanced R&D capability. Under a CS contract, the expected profits of the PBS and the supply chain increase with higher R&D subsidies and enhanced R&D capability; however, the NEVM’s expected profit exhibits a U-shaped relationship with R&D subsidies and R&D capability. (3) Compared with centralized decision-making, neither contract achieves the centralized benchmark for the supply chain’s expected profit. However, compared with decentralized decision-making, the CS contract increases the PBS’s R&D investment and expected profit; the RS contract increases the PBS’s R&D investment and expected profit as well as the supply chain’s expected profit. Both contracts can only improve the NEVM’s expected profit under certain conditions. These findings provide valuable theoretical insights and practical implications for decision-making by NEVMs, PBSs, and government agencies.

## 1. Introduction

The rapid development of the industrial economy and excessive energy consumption have led to substantial CO_2_ emissions, which not only directly exacerbate global warming but also indirectly cause extreme weather events such as droughts and heavy rainfall, posing a serious threat to the sustainable development of human society [1–3]. Consequently, how to reduce energy consumption and lower CO_2_ emissions has become a global priority. The advancement of the new energy vehicle (NEV) sector is crucial for tackling global energy issues and lowering carbon emissions [4–6]. According to the 2022 China Automotive Low-Carbon Action Plan report, pure electric vehicles can reduce carbon emissions by 43.4% compared to conventional gasoline vehicles. Projections suggest that by 2060, the full lifecycle carbon emissions of pure electric vehicles could fall to 23 g/km, highlighting substantial potential for emission reduction. Although NEVs are clean products, issues such as insufficient range and slow charging cause “range anxiety” among consumers, severely hindering the development of NEVs [7,8]. Therefore, overcoming the range technology bottleneck and alleviating consumer “range anxiety” has become a key focus for the NEV industry.

As the core component determining the range of NEVs, power battery R&D typically follows two approaches. Major NEV manufacturers (NEVMs) with substantial resources, such as BYD and Tesla, often opt for independent R&D. However, most NEVMs choose cooperative R&D, where the downstream NEVM collaborates with the upstream power battery supplier (PBS) to jointly develop power batteries. For example, in 2023, several NEVMs, including Li Auto and NIO, signed strategic cooperation agreements with CATL for power battery R&D. However, during cooperative R&D for power batteries, conflicts of interest among supply chain firms can pose significant challenges to collaboration stability. Research shows that R&D cost-sharing (CS) contracts, revenue-sharing (RS) contracts, and two-part tariff contracts that constrain battery prices can help improve supply chain profits [9–11], thereby enhancing cooperation stability. However, these studies are based on the assumption that “R&D is certain to succeed,” overlooking the uncertainty inherent in R&D outcomes. In reality, power battery technology, as a high-tech field, is characterized by high risk, high investment, and long cycles [12,13], and R&D outcomes are often uncertain—the risk of failure always exists. Yang et al. [14] used an exogenous probability to characterize R&D outcome uncertainty. Wang et al. [15] and Chen et al. [16] characterized R&D outcome uncertainty using R&D effort level and R&D investment, respectively. In practice, R&D outcomes are influenced by multiple factors such as R&D investment, R&D capability, and R&D difficulty [17,18]. Considering only a single factor thus fails to comprehensively characterize R&D outcome uncertainty.

Notably, to encourage enterprises to engage in green technology R&D, governments often implement preferential policies such as R&D subsidies. For instance, in 2024, the U.S. Department of Energy allocated $43 million to support R&D for several battery-related technologies, including lithium batteries. In 2025, the Chinese government will restart the solid-state battery major R&D initiative and, for the first time, provide a 15% actual investment subsidy to relevant enterprises through ultra-long-term special government bonds. Research suggests that R&D subsidies can incentivize firms to expand R&D investment [19–22]. However, some scholars argue that R&D subsidies may reduce firms’ own R&D investment, creating a crowding-out effect [23–25]. Additionally, Sun et al. [26], He et al. [27], and Wu et al. [28] posit that the effectiveness of R&D subsidies is conditional. Therefore, whether government R&D subsidy policies have an incentivizing effect on power battery R&D requires further verification. Thus, studying cooperative R&D contracts for NEV power batteries under government subsidies and R&D outcome uncertainty is of significant importance for achieving green development in the supply chain.

Based on the above, considering government subsidies and R&D outcome uncertainty, this paper focuses on a supply chain consisting of a single NEVM and a single PBS. Using an exponential function to characterize R&D outcome uncertainty, it investigates the impacts of R&D subsidies and R&D capability on R&D investment, supply chain members, and their expected profits, and conducts a comparative analysis of CS and RS contracts. The paper addresses the following questions:

(1) From the perspective of firm decision-making, how can R&D outcome uncertainty be characterized endogenously? Considering R&D outcome uncertainty, how does the PBS determine the optimal R&D investment and optimal wholesale price? How does the NEVM determine the sales price to maximize profit?
(2) From the perspective of policy effectiveness, do R&D subsidies generate an incentive effect or a crowding-out effect? What is their impact on R&D investment, supply chain members, and their profits?
(3) Concerning supply chain coordination, what are the impacts of CS and RS contracts on supply chain members and their profits? Can these contracts coordinate supply chain profits? Which contract is preferable? How should the PBS and NEVM select a contract to maximize their respective profits?

The marginal contributions of this paper are threefold:

(1) Based on the industry practice that power batteries can only be produced and sold after successful R&D, this study focuses on the uncertainty of power battery R&D outcomes. It constructs a game model between the NEVM and the PBS, simultaneously examining the influence of R&D capability and government subsidies on supply chain members’ decisions. The results can help supply chains better cope with challenges posed by R&D uncertainty.
(2) Regarding the characterization of R&D outcome uncertainty, differing from the exogenous characterization in existing research, this paper treats R&D outcome uncertainty as an endogenous variable, comprehensively characterizing it from three aspects: R&D investment, R&D difficulty, and R&D capability. It particularly analyzes the impacts of R&D subsidies and R&D capability on R&D investment, the supply chain, and its members’ profits. This enriches the research perspective and addresses gaps in the existing literature.
(3) Our research analyzes the impact of contract forms on cooperative innovation between the NEVM and the PBS. By introducing CS and RS contracts to optimize supply chain performance, it analyzes the differential effects of these two contract types, providing new insights for designing effective coordination contracts for cooperative innovation between NEVMs and PBSs.

The paper proceeds as follows: Section 2 reviews relevant literature; Section 3 outlines the model formulation and solution; Section 4 presents model comparisons and parameter analysis; Section 5 provides numerical simulations; Section 6 offers discussion; and Section 7 concludes.

## 2. Literature review

This study is related to the following streams of literature. First, this study relates to firm innovation decisions under R&D subsidies. This literature primarily focuses on two aspects: First, the impact of R&D subsidies on firm R&D investment. Guellec and Van [19], Pingfang and Weimin [20], and Choi and Lee [21] argue that R&D subsidies can incentivize firms to expand R&D investment. Yao and Zhu [22] used empirical methods to demonstrate that fiscal subsidies significantly promote firm innovation investment. However, some scholars argue that R&D subsidies may reduce firms’ own R&D investment, creating a crowding-out effect [23,25]. An et al. [24] pointed out that firms may obtain government subsidies by releasing false R&D signals that are costly to verify, resulting in subsidies failing to genuinely incentivize R&D. Zhang et al. [29] found that government innovation subsidies have an insignificant impact on firm R&D activities. Additionally, Sun et al. [26] and He et al. [27] argued that the effectiveness of R&D subsidies is conditional. Wu et al. [28] indicated an inverted U-shaped relationship between government subsidy amount and firm R&D investment. In summary, the impact of government R&D subsidies on firm R&D investment remains highly contentious, warranting further verification. Second, the impact of R&D subsidies on the supply chain. Cao et al. [30] and Li et al. [31] found that government subsidies can incentivize green technology innovation behaviors among supply chain members. Chemama et al. [32], Cohen et al. [33], Zhang and Huang [34], and Ju et al. [35] studied how different R&D subsidy methods significantly affect suppliers’ production and pricing decisions. Zhu et al. [36] found that R&D subsidies always increase supply chain members’ profits and consumer surplus in the absence of competition; however, under downstream competition, R&D subsidies are not always beneficial to the manufacturer. Yan et al. [37] stated that under any government subsidy policy, the supply chain can achieve maximum total profit. However, Chen and Sun [38] argued that supply chain profit is maximized only under a centralized decision-making model. The aforementioned literature provides a reference for this study.

Second, this study relates to R&D outcome uncertainty. R&D outcome uncertainty refers to the possibility that R&D outcomes can be either successful or unsuccessful [15]. R&D is typically characterized by high risk, high investment, and long cycles [12,13]. Coupled with the fact that firms cannot fully control all R&D processes, the possibility of R&D failure always exists [18]. Therefore, how to conduct R&D investment under uncertainty becomes particularly important for firm decision-makers. Considering R&D outcome uncertainty, Zhang et al. [39] studied the impact of the degree of cooperative R&D between a supplier and a manufacturer on supply chain members’ profits. Yan and Yang [40] showed that the likelihood of R&D success plays a key role in determining the optimal licensing strategy. In practice, when R&D outcome uncertainty is high, manufacturers often refuse to engage in R&D or choose resource cooperation strategies to obtain more external support [14,41]. Sun et al. [42] studied the impact of manufacturer R&D capability uncertainty on green innovation mode selection. Although the aforementioned literature characterizes R&D outcome uncertainty using the probability of R&D success, these studies treat the success probability as an exogenous variable, overlooking endogeneity issues. Wang et al. [15] considered R&D outcome uncertainty when studying the optimal technology licensing strategy choice for an R&D-oriented manufacturer. Chen et al. [16] studied channel mode selection for manufacturers and retailers selling on traditional online platforms versus blockchain platforms when green technology R&D outcomes are uncertain. Wang and Zhang [43] considered a capital-constrained supplier with R&D failure risk engaging in green product R&D, analyzing the optimal decisions and profits of both supply chain parties under green credit and prepayment financing, respectively. The aforementioned literature treats R&D outcome uncertainty as an endogenous variable related to R&D investment or R&D effort. However, considering only one influencing factor fails to comprehensively characterize R&D outcome uncertainty.

Third, this study relates to supply chain cooperation contracts. In practice, CS and RS contracts are widely used in both industry and academia. In 2018, BMW signed a $117 million agreement with CATL to jointly bear the innovation costs of developing a new generation of battery technology [44]. To promote green technology innovation cooperation between upstream and downstream firms in green supply chains, Jiang and Li [45] and Xu et al. [46] designed CS, RS, and two-part tariff contracts to coordinate the green supply chain. Yenipazarli [47] analyzed the role of CS and RS contracts in promoting corporate green cost reduction. Sharma and Jain [48] studied the optimality of CS contracts in supply chains. Dai et al. [49] and Xu [50] argued that CS contracts bring more profit to supply chain members and their profits. Bai et al. [51] found that CS, RS, and two-part pricing contracts can all achieve perfect coordination, but the two-part pricing contract is more robust. Ma and Guo [9] argued that CS contracts with price constraints are more effective for cooperative R&D in the industry chain. Wu and Zhang [52] argued that CS and profit-sharing contracts can effectively coordinate supply chain members to achieve Pareto efficiency. Cai et al. [53] found that both CS contracts and option contracts can achieve Pareto improvements in supply chain performance under certain conditions. The aforementioned literature provides references for the cooperative R&D contract design in this paper.

Finally, the literature most closely related to this study includes Ghosh and Shah [54], Wang et al. [55], Zhou et al. [56], Wang [11], and Zheng et al. [57]. Ghosh and Shah [54] explored the impact of CS contracts on green supply chain participants’ decisions. Wang et al. [55] studied contract design for joint emission reduction between a manufacturer and a retailer in a supply chain. Zhou et al. [56], focusing on a retailer-dominated bilateral monopoly green supply chain, studied the impact of CS contracts on green product demand, supply chain member profits, and channel profit. Considering green R&D subsidy policies, Wang [11] studied the impact of CS and RS contracts on the supply chain. Zheng et al. [57] studied a two-echelon supply chain comprising a manufacturer and a retailer selecting carbon neutrality contracts to address net-zero carbon emission production. The aforementioned literature constructs models based on the premise of R&D success, overlooking R&D outcome uncertainty. However, differing from their research, this paper is based on the reality of R&D outcome uncertainty and uses an exponential function to characterize the probability of R&D success from three aspects: R&D capability, R&D investment, and R&D difficulty (range capability). Furthermore, differing from the conclusions of Ghosh and Shah [54] and Zheng et al. [57], we find that CS and RS contracts can only improve the downstream manufacturer’s profit under certain conditions. Wang [11] argued that RS contracts can improve overall supply chain profit and enhance social welfare levels. However, we find that neither CS nor RS contracts can achieve the centralized benchmark of supply chain expected profit [55,56]. Notably, we also find an inverted U-shaped relationship between R&D capability and R&D investment, which is not mentioned in the aforementioned literature.

Existing literature provides a foundation but requires refinement. Table 1 shows a comparison with selected literature. On one hand, regarding the characterization of R&D outcome uncertainty, existing literature either uses exogenous variables or a single endogenous variable (R&D investment or R&D effort) for characterization. However, in practice, factors such as firm R&D capability and R&D difficulty also significantly influence R&D outcome uncertainty. Therefore, it is necessary to comprehensively consider the impacts of R&D investment, R&D capability, and R&D difficulty on R&D outcome uncertainty. On the other hand, existing literature focuses on the impact of government subsidies on supply chain coordination mechanisms, and research conclusions remain highly divergent. There is limited literature on designing cooperative R&D contracts for power batteries from an industry chain perspective, considering government subsidies and R&D outcome uncertainty. Therefore, further analysis of the specific impacts of CS and RS contracts on the industry chain is warranted.

**Table 1:** Comparison with selected literature.

Literature	Subsidy	R&D Uncertainty	CS Contract	RS Contract
Ma and Guo [9]	√	×	√	×
Zhou et al. [56]	×	×	√	×
Wang [11]	√	×	√	√
Ghosh and Shah [54]	×	×	√	×
Sun et al. [42]	×	Exogenous	√	√
Liu et al. [41]	×	Exogenous	×	√
Wang et al. [55]	×	Endogenous	×	×
Zhu et al. [36]	√	×	×	×
Yan et al. [37]	√	×	√	×
Wu and Zhang [52]	√	×	√	√
Dai et al. [49]	√	×	√	×
This Paper	√	Endogenous	√	√

## 3. Model formulation and solution

### 3.1 Basic assumptions

This paper focuses on a supply chain consisting of a single NEVM and a single PBS. Both firms are risk-neutral and aim to maximize expected profit. Considering government subsidies and R&D outcome uncertainty, the two parties engage in R&D activities to enhance power battery range capability by signing a cooperation contract. The NEVM plans to increase the power battery range capability from *b*_0_ to *b* (*b* > *b*_0_). Let $\Delta_{b}={b-b}_{\text{0}}$, where $\Delta_{b}$ denotes the target increment in range capability and also serves as a proxy for R&D difficulty. A larger target range improvement generally implies a more difficult R&D task. Therefore, for given I and λ, a larger $\Delta_{b}$ lowers the probability of R&D success. The PBS is responsible for battery R&D. Typically, power batteries undergo three stages from R&D to sale: the R&D stage, the production stage, and the sales stage. After successful R&D, the battery enters the production stage (production cost *c*) and is sold to the NEVM at wholesale price *w*. Subsequently, the NEVM produces the NEV and sells it to consumers. If R&D fails, no power battery is produced or sold, and both the NEVM and the PBS receive no sales revenue. R&D investment costs are incurred during the R&D stage regardless of success or failure. To enhance scientific rigor, the following descriptions and assumptions are made:

**Assumption 1** In the R&D stage, the R&D investment is *I* > 0, and the government provides a corresponding R&D subsidy with a coefficient η∈(0,1). The government subsidy ηI is paid when R&D investment is made, regardless of whether R&D succeeds.

**Assumption 2** Power battery R&D outcomes are uncertain, i.e., R&D may succeed or fail. This paper uses the probability of R&D success (f) to characterize R&D outcome uncertainty. Based on research by Huang et al. [58], Yu and Liu [59], and Chen et al. [16], this paper employs an exponential function to characterize the R&D success probability. Assume *f* is positively correlated with R&D capability (λ>0) and R&D investment (I), and negatively correlated with R&D difficulty ($\Delta_{b}$), i.e., $f=\text{1}-\text{exp}(-\lambda I/\Delta_{b})$.

The exponential form $f=\text{1}-\text{exp}\left(-\lambda I/\Delta_{b}\right)$ is adopted for the following reasons. First, if I=0, then f=0, meaning that R&D cannot succeed without investment. Second, f increases with R&D investment I, i.e., ∂f/∂I>0. Third, f increases with R&D capability λ, i.e., ∂f/∂λ>0. Fourth, f decreases with R&D difficulty $\Delta_{b}$, i.e., $\partial f/\partial \Delta_{b}<\text{0}$. Fifth, 0≤f<1, so the probability is bounded. Sixth, $\partial^{\text{2}}f/\partial I^{\text{2}}<\text{0}$, which captures diminishing marginal returns to R&D investment. The term $\lambda I/\Delta_{b}$ can be interpreted as effective R&D effort per unit of R&D difficulty, or R&D investment efficiency adjusted by capability and difficulty. This functional form is therefore consistent with high-risk power battery R&D, where additional investment raises the success probability but with diminishing marginal effects.

**Assumption 3** Drawing on research by Ma and Guo [9] and Zhou et al. [56], in the sales stage, the NEVM faces the demand function $Q=A-\alpha p+\beta \Delta_{b}$, where *A* represents potential market demand; *p* represents the NEV sales price; *α* > 0 represents the price sensitivity coefficient; and *β* > 0 represents consumers’ sensitivity coefficient to range capability improvement.

Furthermore, to ensure the existence and non-negativity of equilibrium solutions, assume $exp(-\lambda I/\Delta_{b})<\text{2}UV/(U^{\text{2}}+V^{\text{2}})$, where $U=(\text{2}-s)\left[(\text{1}-s)\left(A+\beta \Delta_{b}\right)-\alpha w\right],V=s(\text{1}-s)\left(A+\beta \Delta_{b}\right)+(\text{2}-s)\alpha w-\text{2}(\text{1}-s)\alpha c$. Also assume $exp(-\lambda I/\Delta_{b})<\text{2}\alpha (x-c)\left(A-\alpha x+\beta \Delta_{b}\right)/[{\left(A-\alpha x+\beta \Delta_{b}\right)}^{\text{2}}+\alpha^{\text{2}}(x-c{)}^{\text{2}}]$, where x∈{p,w}. Let superscript N = {DD, CD, CS, RS} denote decentralized decision-making, centralized decision-making, CS contract, and RS contract, respectively. Subscripts 1, 2, and T denote the NEVM, PBS, and the supply chain system, respectively. Symbols and descriptions are listed in Table 2.

**Table 2:** Model symbols and descriptions.

Symbol	Description	Symbol	Description
$b_{\text{0}}$	Initial range capability	$\Delta_{b}$	Range capability increment
b	Target range capability	Q	Market demand for NEVs
*p*	NEV price	*A*	Potential market demand
*w*	Battery wholesale price	f	Probability of R&D success
*I*	R&D investment	t	Proportion of R&D cost borne by NEVM
λ	R&D capability	η	Government R&D subsidy ratio
π	Expected profit	*s*	Proportion of operating revenue received by PBS
*c*	Battery production cost	α	Price sensitivity coefficient
β	Consumers’ sensitivity coefficient to range capability improvement		

### 3.2 Decentralized decision-making

As the baseline, under decentralized decision-making, the battery R&D cost is entirely borne by the PBS, and the NEVM purchases batteries from the PBS only at wholesale price *w*. In this case, the PBS first chooses *w* and *I*, and then the NEVM chooses *p*. Therefore, the expected profits of the NEVM and PBS are respectively,


$$\begin{array}{c} \begin{array}{c} \pi_{\text{1}}^{DD}=\left[\text{1}-exp(-\lambda I/\Delta_{b})\right](p-w)\left(A-\alpha p+\beta \Delta_{b}\right) \end{array} \end{array}$$ (1)

$$\begin{array}{c} \begin{array}{c} \pi_{\text{2}}^{DD}=\left[\text{1}-exp(-\lambda I/\Delta_{b})\right](w-c)\left(A-\alpha p+\beta \Delta_{b}\right)-(\text{1}-\eta )I \end{array} \end{array}$$ (2)

Using backward induction to solve, Proposition 1 is obtained.

**Proposition 1:** Under decentralized decision-making, the optimal sales price is $p^{DD*}$, the maximum market demand is $Q^{DD*}$, the optimal wholesale price is $w^{DD*}$, the optimal R&D investment is $I^{DD*}$, the NEVM’s maximum expected profit is $\pi_{\text{1}}^{DD*}$, the PBS’s maximum expected profit is $\pi_{\text{2}}^{DD*}$, and the supply chain’s maximum expected profit is $\pi_{T}^{DD*}$.

**Proof:** See A.1 in S1 Appendix.

Table 3 summarizes the equilibrium solutions under decentralized decision-making as well as the centralized decision-making, CS contract, and RS contract models. Here, $H=A+\beta \Delta_{b}-\alpha c,K=(\text{1}-\eta )\Delta_{b}/\lambda$.

**Table 3:** Equilibrium solutions under different models.

Equilibrium solution	Decentralized Decision	Centralized Decision	CS Contract	RS Contract
$p^{(\;)*\;}$	$\frac{\text{3}\left(A+\beta \Delta_{b}\right)+\alpha c}{\text{4}\alpha }$	$\frac{A+\beta \Delta_{b}+\alpha c}{\text{2}\alpha }$	$\frac{\text{3}\left(A+\beta \Delta_{b}\right)+\alpha c}{\text{4}\alpha }$	$\frac{(\text{3}-\text{2}s)\left(A+\beta \Delta_{b}\right)+\alpha c}{\text{2}\alpha (\text{2}-s)}$
$Q^{(\;)*\;}$	$\frac{H}{\text{4}}$	$\frac{H}{\text{2}}$	$\frac{H}{\text{4}}$	$\frac{H}{\text{2}(\text{2}-s)}$
$w^{(\;)*\;}$	$\frac{A+\beta \Delta_{b}+\alpha c}{\text{2}\alpha }$	——	$\frac{A+\beta \Delta_{b}+\alpha c}{\text{2}\alpha }$	$\frac{(\text{1}-s{)}^{\text{2}}\left(A+\beta \Delta_{b}\right)+\alpha (\text{1}-s)c}{\alpha (\text{2}-s)}$
$I^{(\;)*\;}$	$\frac{\Delta_{b}}{\lambda }\;ln\frac{H^{\text{2}}}{\text{8}\alpha K}$	$\frac{\Delta_{b}}{\lambda }ln\frac{H^{\text{2}}}{\text{4}\alpha K}$	$\frac{\Delta_{b}}{\lambda }\;ln\frac{H^{\text{2}}}{\text{8}\alpha (\text{1}-t)K}$	$\frac{\Delta_{b}}{\lambda }\;ln\frac{H^{\text{2}}}{\text{4}\alpha (\text{2}-s)K}$
$\pi_{\text{1}}^{(\;)*}$	$\frac{H^{\text{2}}}{\text{1}\text{6}\alpha }-\frac{K}{\text{2}}$	——	$\frac{H^{\text{2}}}{\text{1}\text{6}\alpha }-\frac{(\text{1}-t)K}{\text{2}}-tK\;ln\frac{H^{\text{2}}}{\text{8}\alpha (\text{1}-t)K}$	$\frac{(\text{1}-s)H^{\text{2}}}{\text{4}\alpha (\text{2}-s{)}^{\text{2}}}-\frac{(\text{1}-s)K}{\text{2}-s}$
$\pi_{\text{2}}^{(\;)*}$	$\frac{H}{\text{8}\alpha }-K-K\;ln\frac{H^{\text{2}}}{\text{8}\alpha K}$	——	$\frac{H^{\text{2}}}{\text{8}\alpha }-(\text{1}-t)K-(\text{1}-t)K\;ln\frac{H^{\text{2}}}{\text{8}\alpha (\text{1}-t)K}$	$\frac{H^{\text{2}}}{\text{4}\alpha (\text{2}-s)}-K-K\;ln\frac{H}{\text{4}\alpha (\text{2}-s)K}$
$\pi_{T}^{(\;)*}$	$\frac{\text{3}H^{\text{2}}}{\text{1}\text{6}\alpha }-\frac{\text{3}K}{\text{2}}-K\;ln\frac{H^{\text{2}}}{\text{8}\alpha K}$	$\frac{H^{\text{2}}}{\text{4}\alpha }-K-Kln\frac{H^{\text{2}}}{\text{4}\alpha K}$	$\frac{{\text{3}H}^{\text{2}}}{\text{1}\text{6}\alpha }-\frac{\text{3}(\text{1}-t)K}{\text{2}}-K\;ln\frac{H^{\text{2}}}{\text{8}\alpha (\text{1}-t)K}$	$\frac{(\text{3}-\text{2}s)H^{\text{2}}}{\text{4}\alpha (\text{2}-s{)}^{\text{2}}}-\frac{(\text{3}-\text{2}s)K}{\text{2}-s}-K\;ln\frac{H^{\text{2}}}{\text{4}\alpha (\text{2}-s)K}$

It should be noted that, to ensure interior and non-negative equilibrium solutions, the following parameter restrictions are imposed. First, $A+\beta \Delta_{b}-\alpha c>\text{0}$, which ensures positive market demand. Second, to ensure that the equilibrium solutions for I, p, and w are non-negative, we require $\frac{\lambda {\left(A+\beta \Delta_{b}-\alpha c\right)}^{\text{2}}}{\text{8}\alpha (\text{1}-\eta )\Delta_{b}}>\text{1}\;$for decentralized and centralized decision-making; $\frac{\lambda {\left(A+\beta \Delta_{b}-\alpha c\right)}^{\text{2}}}{\text{8}\alpha (\text{1}-t)(\text{1}-\eta )\Delta_{b}}>\text{1}\;$for the CS contract; $\frac{\lambda {\left(A+\beta \Delta_{b}-\alpha c\right)}^{\text{2}}}{\text{4}\alpha (\text{2}-s)(\text{1}-\eta )\Delta_{b}}>\text{1}$ for the RS contract.

### 3.3 Centralized decision-making

Under centralized decision-making, the NEVM and PBS in the supply chain are treated as a single entity. At this point, a central planner chooses p and I to maximize total supply chain expected profit. Therefore, the supply chain’s expected profit $\pi_{T}^{CD}$ is,


$$\begin{array}{c} \begin{array}{c} \pi_{T}^{CD}=\left[\text{1}-exp(-\lambda I/\Delta_{b})\right](p-c)\left(A-\alpha p+\beta \Delta_{b}\right)-(\text{1}-\eta )I \end{array} \end{array}$$ (3)

Using backward induction to solve, Proposition 2 is obtained.

**Proposition 2:** Under centralized decision-making, the optimal sales price is $p^{CD*}$, the maximum market demand is $Q^{CD*}$, the optimal R&D investment is $I^{CD*}$, and the supply chain’s maximum expected profit is $\pi_{T}^{CD*}$.

**Proof:** See A.2 in S1 Appendix=.

### 3.4 Supply chain coordination contracts

#### 3.4.1 CS contract.

The CS contract is a widely adopted contract form in supply chains. Under this contract, the PBS sets the battery wholesale price w, and the NEVM pays w for each battery. The NEVM bears a proportion t of the after-subsidy R&D investment cost, while the PBS bears the remaining proportion 1−t, where t∈(0,1). It should be noted that the CS ratio t is either exogenously given or determined through negotiation before operational decisions are made; it is not a decision variable in the model. In the CS case, *t* is predetermined; the PBS chooses *w* and *I*, and the NEVM chooses *p*. Therefore, expected profits are,


$$\begin{array}{c} \begin{array}{c} \pi_{\text{1}}^{CS}=\left[\text{1}-exp(-\lambda I/\Delta_{b})\right](p-w)\left(A-\alpha p+\beta \Delta_{b}\right)-t(\text{1}-\eta )I \end{array} \end{array}$$ (4)

$$\begin{array}{c} \begin{array}{c} \pi_{\text{2}}^{CS}=\left[\text{1}-exp(-\lambda I/\Delta_{b})\right](w-c)\left(A-\alpha p+\beta \Delta_{b}\right)-(\text{1}-t)(\text{1}-\eta )I \end{array} \end{array}$$ (5)

Using backward induction to solve, Proposition 3 is obtained.

**Proposition 3:** Under the CS contract, the optimal sales price is $p^{CS*}$, the maximum market demand is $Q^{CS*}$, the optimal wholesale price is $w^{CS*}$, the optimal R&D investment is $I^{CS*}$, the NEVM’s maximum expected profit is $\pi_{\text{1}}^{CS*}$, the PBS’s maximum expected profit is $\pi_{\text{2}}^{CS*}$, and the supply chain’s maximum expected profit is $\pi_{T}^{CS*}$.

**Proof:** Similar to Proposition 1, thus omitted.

#### 3.4.2 RS contract.

Under the RS contract, the NEVM purchases batteries at price *w* and transfers a portion of its revenue (*s*) to the PBS. It should be noted that the RS ratio s is either exogenously given or determined through negotiation before operational decisions are made; it is not a decision variable in the model. In the RS case, *s* is predetermined; the PBS chooses *w* and *I*, and the NEVM chooses *p*. Therefore, the expected profits of the NEVM and PBS are respectively,


$$\begin{array}{c} \begin{array}{c} \pi_{\text{1}}^{RS}=\left[\text{1}-exp(-\lambda I/\Delta_{b})\right]\left[(\text{1}-s)p-w\right]\left(A-\alpha p+\beta \Delta_{b}\right) \end{array} \end{array}$$ (6)

$$\begin{array}{c} \begin{array}{c} \pi_{\text{2}}^{RS}=\left[\text{1}-exp(-\lambda I/\Delta_{b})\right](sp+w-c)\left(A-\alpha p+\beta \Delta_{b}\right)-(\text{1}-\eta )I \end{array} \end{array}$$ (7)

Using backward induction to solve, Proposition 4 is obtained.

**Proposition 4:** Under the RS contract, the optimal sales price is $p^{RS*}$, the maximum market demand is $Q^{RS*}$, the optimal wholesale price is $w^{RS*}$, the optimal R&D investment is $I^{RS*}$, the NEVM’s maximum expected profit is $\pi_{\text{1}}^{RS*}$, the PBS’s maximum expected profit is $\pi_{\text{2}}^{RS*}$, and the supply chain’s maximum expected profit is $\pi_{T}^{RS*}$.

**Proof**: Similar to Proposition 1, thus omitted.

## 4. Equilibrium analysis

This section examines: (1) the impact of R&D capability λ and subsidy coefficient η on R&D investment and expected profits; (2) a comparison of R&D investment and profits under centralized and decentralized decision-making.

### 4.1 Sensitivity analysis

**Proposition 5:**

①$\left\{ \begin{array}{c} \frac{\partial I^{N*}}{\partial \lambda }>\text{0},\;if\;\text{0}<\lambda <\lambda^{N*}\\ \frac{\partial I^{N*}}{\partial \lambda }<\text{0},\;if\;\lambda >\lambda^{N*}\; \end{array}\right.\;$.
②$\frac{\partial \pi_{\text{1}}^{DD*}}{\partial \lambda }>\text{0}$,$\;\frac{\partial \pi_{\text{1}}^{RS*}}{\partial \lambda }>\text{0}$.
③$\left\{ \begin{array}{c} \frac{\partial \pi_{\text{1}}^{CS}}{\partial \lambda }>\text{0},\;if\;\frac{\text{1}-\text{3}t}{\text{2}}+tln\frac{H^{\text{2}}}{\text{8}\alpha (\text{1}-t)K}>\text{0}\\ \;\frac{\partial \pi_{\text{1}}^{CS}}{\partial \lambda }<\text{0},\;if\;\frac{\text{1}-\text{3}t}{\text{2}}+tln\frac{H^{\text{2}}}{\text{8}\alpha (\text{1}-t)K}<\text{0} \end{array}\right.\;$.
④$\frac{\partial \pi_{\text{2}}^{DD*}}{\partial \lambda }>\text{0}$, $\frac{\partial \pi_{\text{2}}^{CS*}}{\partial \lambda }>\text{0}$, $\frac{\partial \pi_{\text{2}}^{RS*}}{\partial \lambda }>\text{0}$.
⑤$\frac{\partial \pi_{T}^{N*}}{\partial \lambda }>\text{0}$.

**Proof:** See A.3 in S1 Appendix.

Proposition 5 analyzes the impact of R&D capability. Conclusion ① indicates an inverted U-shaped relationship between the PBS’s R&D capability and its R&D investment [28]. This differs from the “positive relationship between R&D capability and R&D investment” conclusion proposed by Liu et al. [60]. Specifically, when the PBS’s R&D capability is weak, increasing R&D investment helps overcome technical bottlenecks and improves the probability of R&D success, thereby reducing R&D outcome uncertainty and increasing marginal returns. Therefore, R&D investment increases with R&D capability. However, interestingly, when R&D capability surpasses a certain threshold ($\lambda^{N*}$), further enhancement leads to reduced R&D investment by the PBS. The reason is that when λ is sufficiently high, the PBS can achieve a relatively high probability of success with less investment, so the optimal R&D investment may decline. Hence, I and λ exhibit an inverted U-shaped relationship. Descriptive evidence from LG Chem’s R&D expenditure during 2014–2019 provides a suggestive empirical pattern that is broadly consistent with the inverted U-shaped relationship identified in the model. This empirical data further verifies conclusion ①.

Conclusion ② indicates that under decentralized decision-making and the RS contract, the NEVM’s expected profit increases with R&D capability. Enhanced R&D capability generates two effects: direct and indirect. When R&D capability is low, its enhancement stimulates the PBS to increase R&D investment, further improving R&D success probability and reducing outcome uncertainty, directly leading to increased expected profit [10,61], i.e., a direct effect. However, differing from Ma and Guo [61], when R&D capability is high, although its enhancement reduces R&D investment, the technology is more mature, unit R&D investment efficiency is higher, and cost savings outweigh revenue loss, indirectly leading to increased expected profit, i.e., an indirect effect. Under decentralized decision-making and the RS contract, the NEVM does not directly bear R&D costs. Therefore, under the combined direct and indirect effects, the NEVM’s expected profit increases.

Conclusion ③ indicates that under the CS contract, under certain conditions, the NEVM’s expected profit increases with R&D capability. This differs from Wang [11], who concluded that “CS contracts reduce downstream retailer profit.” Under the CS contract, the NEVM bears a portion of the R&D cost. Although the direct and indirect effects cause the NEVM’s expected sales profit to increase, its share of R&D cost also increases. Notably, when the NEVM’s CS ratio *t* is too large, it further stimulates increased R&D cost. When the increase in the NEVM’s R&D cost share exceeds the increase in expected sales profit, its expected profit decreases with enhanced R&D capability.

Conclusion ④ indicates that the PBS’s expected profit increases with R&D capability [42]. Under decentralized decision-making, the direct and indirect effects of enhanced capability directly increase the PBS’s expected profit. However, under CS and RS contracts, the NEVM also directly or indirectly compensates the PBS’s R&D cost, reducing the PBS’s R&D cost burden, creating a cost compensation effect. Under decentralized decision-making and the CS contract, the PBS’s expected operating profit is (w−c)Q. Under RS, it is (sp+w−c)Q. Therefore, under the combined direct, indirect, and cost compensation effects, the PBS’s expected sales profit exceeds its R&D cost, ultimately leading to increased expected profit with enhanced R&D capability.

Conclusion ⑤ indicates that the supply chain’s expected profit increases with PBS R&D capability. This aligns with the conclusion of Ma and Guo [61]. As described, under the direct and indirect effects, the increase in the supply chain’s expected sales profit exceeds the additional R&D cost incurred, leading to increased supply chain expected profit.

**Proposition 6:**

①$\frac{\partial I^{N*}}{\partial \eta }>\text{0}$.
②$\frac{\partial \pi_{\text{1}}^{DD*}}{\partial \eta }>\text{0}$, $\frac{\partial \pi_{\text{1}}^{RS*}}{\partial \eta }>\text{0}$.
③$\left\{ \begin{array}{c} \frac{\partial \pi_{\text{1}}^{CS}}{\partial \eta }>\text{0},\;if\;\frac{\text{1}-\text{3}t}{\text{2}}+tln\frac{H^{\text{2}}}{\text{8}\alpha (\text{1}-t)K}>\text{0}\\ \;\frac{\partial \pi_{\text{1}}^{CS}}{\partial \eta }<\text{0},\;if\;\frac{\text{1}-\text{3}t}{\text{2}}+tln\frac{H^{\text{2}}}{\text{8}\alpha (\text{1}-t)K}<\text{0} \end{array}\right.\;.$
④$\frac{\partial \pi_{\text{2}}^{DD*}}{\partial \eta }>\text{0}$, $\frac{\partial \pi_{\text{2}}^{CS*}}{\partial \eta }>\text{0}$, $\frac{\partial \pi_{\text{2}}^{RS*}}{\partial \eta }>\text{0}$.
⑤$\frac{\partial \pi_{T}^{N*}}{\partial \eta }>\text{0}$.

**Proof:** The proof method is similar to Proposition 5, thus omitted.

Proposition 6 analyzes the impact of R&D subsidies. Conclusion ① indicates that government R&D subsidies have an incentive effect: as government R&D subsidies increase, PBS R&D investment increases. This aligns with the conclusions of Guellec and Van [19], Pingfang and Weimin [20], Choi and Lee [21], and Yao and Zhu [22]. Government subsidies directly reduce the PBS’s marginal cost, lowering the “threshold” for R&D investment, making firms more willing to increase investment to capture the expected revenue from a higher success probability. Specifically, without subsidies, supply chain firms bear all R&D costs. Due to outcome uncertainty, firms may reduce investment to avoid losses. However, government subsidies effectively share part of the PBS’s R&D cost (subsidy ratio *η*), reducing the potential loss from failure and incentivizing firms to increase investment to pursue higher success probability. Therefore, increased subsidies lower the PBS’s effective R&D cost, increasing the marginal net return on R&D investment and incentivizing the PBS to increase investment.

Conclusion ② indicates that under decentralized decision-making and the RS contract, the NEVM’s expected profit increases with higher government R&D subsidies. Increased R&D subsidies have two effects: a direct effect, where subsidies directly reduce power battery R&D cost; and an indirect effect, where the incentive effect of subsidies stimulates the PBS to increase R&D investment, thereby improving R&D success probability and reducing outcome uncertainty. Under decentralized decision-making and the RS contract, R&D costs are directly borne by the PBS. Due to the indirect effect of subsidies, the NEVM’s expected profit increases as higher subsidies.

Conclusion ③ indicates that under the CS contract, under certain conditions, the NEVM’s expected profit increases with higher R&D subsidies [37]. As mentioned, R&D subsidies generate direct and indirect effects. When the NEVM’s CS ratio *t* is too large, it further stimulates increased R&D cost. That is, the indirect effect of subsidies significantly increases R&D cost, and the NEVM’s share also increases. When the increase in the NEVM’s R&D cost share exceeds the increase in expected sales profit, its expected profit decreases with higher R&D subsidies.

Conclusion ④ indicates that the PBS’s expected profit increases with higher government R&D subsidies. As mentioned, under the direct and indirect effects of subsidies, as subsidies increase, the increase in the PBS’s expected revenue exceeds the increase in its R&D cost share, so its expected profit increases.

Conclusion ⑤ indicates that the supply chain’s expected profit increases with higher government R&D subsidies [62]. This conclusion differs from Cao et al. [63], who proposed an “inverted U-shaped relationship between government subsidies and supply chain profit.” Without government subsidies, power battery R&D costs are entirely borne by supply chain firms, exposing them to the risk of loss from failure. Without government subsidies, firms bear the full R&D cost and are exposed to potential losses if R&D fails. Under expected-profit maximization, this failure exposure reduces the marginal attractiveness of additional R&D investment. However, with government subsidies, due to their direct and indirect effects, R&D success probability increases and outcome uncertainty decreases, leading to increased supply chain expected profit with higher subsidies.

### 4.2 Comparative analysis

**Proposition 7:**

①$I^{CS*}>I^{DD*}$, $I^{CD*}>I^{RS*}>I^{DD*}$.
②$\left\{ \begin{array}{c} I^{CD*}>I^{CS*},\;if\;\text{0}<t<\text{0}.\text{5}\\ I^{CD*}<I^{CS*},\;if\;\text{0}.\text{5}<t<\text{1} \end{array}\right.\;$.
③$\left\{ \begin{array}{c} I^{CS*}>I^{RS*},\;if\;s<\text{2}t\\ I^{CS*}<I^{RS*},\;if\;s>\text{2}t \end{array}\right.\;.$

**Proof:** See A.4 in S1 Appendix.

Proposition 7 analyzes the magnitude of R&D investment under different scenarios. Specifically, conclusion ① indicates that compared with decentralized decision-making, both CS and RS contracts can increase R&D investment. However, the RS contract cannot achieve the optimal R&D investment under centralized decision-making. Under decentralized decision-making, the PBS bears the entire R&D cost alone. The PBS decides R&D investment based on its own expected profit maximization rather than that of the supply chain. Therefore, the PBS tends to reduce R&D investment to avoid losses from potential failure, resulting in the lowest R&D investment. Under CS and RS contracts, the PBS receives direct or indirect compensation for its R&D investment, so $I^{CS*}$ and $I^{RS*}$ are greater than $I^{DD*}$.

Conclusion ② indicates that compared to centralized decision-making, influenced by the CS ratio *t*, the CS contract can increase power battery R&D investment under certain conditions [9]. When the NEVM bears a small portion of R&D cost (0 < *t* < 0.5), the PBS bears most of the cost. To avoid losses from potential failure, the PBS may reduce investment. Therefore, $I^{CD*}>I^{CS*}$. However, when the NEVM bears a larger portion (0.5 < *t* < 1), the PBS bears a smaller portion, incentivizing further investment, so $I^{CD*}<I^{CS*}$.

Conclusion ③ indicates that the magnitude of R&D investment under the CS contract versus the RS contract depends on the CS ratio *t* and RS ratio *s*. This differs from Wang [11], who concluded that “R&D investment is larger under the RS contract compared to the CS contract.” When the NEVM bears a large share of R&D cost but receives a small share of revenue (*s* < 2*t*), the CS contract provides stronger incentives for the PBS to increase investment, so $I^{CS*}$>$I^{RS*}$. Conversely, the RS contract provides better incentives.

**Proposition 8:**

①$\left\{ \begin{array}{c} \pi_{\text{1}}^{DD*}>\pi_{\text{1}}^{CS*},\;if\;\frac{H^{\text{2}}}{\text{8}\alpha (\text{1}-t)K}>\sqrt{e}\;\\ \pi_{\text{1}}^{DD*}<\pi_{\text{1}}^{CS*}\;,\;if\;\text{1}<\frac{H^{\text{2}}}{\text{8}\alpha (\text{1}-t)K}<\sqrt{e} \end{array}\right.\;$.
②$\left\{ \begin{array}{c} \pi_{\text{1}}^{DD*}>\pi_{\text{1}}^{RS*},\;if\;\frac{H^{\text{2}}}{\text{8}\alpha K}>\frac{\text{2}-s}{s}\;\\ \pi_{\text{1}}^{DD*}<\pi_{\text{1}}^{RS*},\;if\;\text{1}<\frac{H^{\text{2}}}{\text{8}\alpha K}<\frac{\text{2}-s}{s} \end{array}\right.\;$.
③$\left\{ \begin{array}{c} \pi_{\text{1}}^{CS*}>\pi_{\text{1}}^{RS*},\;if\;\frac{s^{\text{2}}H^{\text{2}}}{{\text{1}\text{6}\alpha (\text{2}-s)}^{\text{2}}}+\left(\frac{\text{1}+t}{\text{2}}-\frac{\text{1}}{\text{2}-s}\right)K-K\;ln\frac{H^{\text{2}}}{\text{8}\alpha (\text{1}-t)K}>\text{0}\;\\ \pi_{\text{1}}^{CS*}<\pi_{\text{1}}^{RS*},\;if\;\frac{s^{\text{2}}H^{\text{2}}}{{\text{1}\text{6}\alpha (\text{2}-s)}^{\text{2}}}+\left(\frac{\text{1}+t}{\text{2}}-\frac{\text{1}}{\text{2}-s}\right)K-K\;ln\frac{H^{\text{2}}}{\text{8}\alpha (\text{1}-t)K}<\text{0} \end{array}\right.\;$.

**Proof:** The proof method is similar to Proposition 7, thus omitted.

Proposition 8 analyzes the magnitude of the NEVM’s expected profit under different scenarios. Conclusion ① indicates that compared with decentralized decision-making, the CS contract can increase the NEVM’s expected profit under certain conditions. This differs from Ghosh and Shah [54], who concluded that “CS contracts increase retailer profit.” Specifically, under both decentralized decision-making and the CS contract, the NEVM’s sales profit (p−w)Q is equal. However, under decentralized decision-making, the NEVM does not bear any R&D cost, so its expected profit depends only on the R&D success probability ($\text{1}-\text{8}\alpha K/H^{\text{2}}$). Under the CS contract, the NEVM must bear part of the R&D cost, so its expected profit depends not only on the success probability ($\text{1}-\text{8}\alpha (\text{1}-t)K/H^{\text{2}}$) but also on its share $tKln(H^{\text{2}}/\text{8}\alpha (\text{1}-t)K)$. Notably, the success probability under decentralized decision-making is lower than under the CS contract. Therefore, when the increase in expected revenue from the higher probability is less than the additional R&D cost borne, i.e., when $H^{\text{2}}>\text{8}\alpha (\text{1}-t)K\sqrt{e}$, then $\pi_{\text{1}}^{DD*}>\pi_{\text{1}}^{CS*}$. Conversely, if the revenue increase exceeds the cost, i.e., $H^{\text{2}}<\text{8}\alpha (\text{1}-t)K\sqrt{e}$, then $\pi_{\text{1}}^{DD*}<\pi_{\text{1}}^{CS*}$.

Conclusion ② indicates that compared with decentralized decision-making, the RS contract can increase the NEVM’s expected profit under certain conditions. This differs from Zheng et al. [57], who concluded that “RS contracts increase retailer profit.” Specifically, under the RS contract, the NEVM transfers part of its revenue to the PBS to compensate for R&D cost. Note that the NEVM’s sales profit ((1−s)p−w)Q under this contract is lower than under decentralized decision-making (p−w)Q. However, the PBS, receiving compensation, increases R&D investment, leading to higher success probability. When $\text{1}<H^{\text{2}}/\text{8}\alpha K<(\text{2}-s)/s$, the increased success probability causes the NEVM’s expected profit to exceed that under decentralized decision-making. However, when $H^{\text{2}}/\text{8}\alpha K>(\text{2}-s)/s$, the probability increase is insufficient to significantly raise the NEVM’s expected profit, so $\pi_{\text{1}}^{DD*}>\pi_{\text{1}}^{RS*}$.

Conclusion ③ indicates that the effectiveness of CS versus RS contracts in improving the NEVM’s expected profit depends on the combined effects of the CS ratio *t* and RS ratio *s*. This differs from Zheng et al. [57], who concluded that “retailer profit is larger under the RS contract.” In fact, when *t* is large or *s* is small, R&D cost $I^{CS*}$ under the CS contract is higher than $I^{RS*}$ under the RS contract. At the same time, the success probability and NEVM’s expected sales profit (p−w)Q are also higher under the CS contract. However, because the NEVM bears a higher proportion of R&D cost, its expected profit is reduced. Therefore, the CS contract is less effective than the RS contract. Conversely, when *s* is large or *t* is small, the CS contract is more effective.

**Proposition 9:**

①$\pi_{\text{2}}^{CS*}>\pi_{\text{2}}^{DD*}$, $\pi_{\text{2}}^{RS*}>\pi_{\text{2}}^{DD*}$.
②$\left\{ \begin{array}{c} \pi_{\text{2}}^{CS*}>\pi_{\text{2}}^{RS*},\;if\;\frac{sH^{\text{2}}}{\text{8}\alpha (\text{2}-s)}-tK+Kln\frac{\left(\text{2}-s\right)}{\text{2}(\text{1}-t)}-tKln\frac{H^{\text{2}}}{\text{8}\alpha (\text{1}-t)K}<\text{0}\\ \pi_{\text{2}}^{CS*}<\pi_{\text{2}}^{RS*},\;if\;\frac{sH^{\text{2}}}{\text{8}\alpha (\text{2}-s)}-tK+Kln\frac{\left(\text{2}-s\right)}{\text{2}(\text{1}-t)}-tKln\frac{H^{\text{2}}}{\text{8}\alpha (\text{1}-t)K}>\text{0} \end{array}\right.$

**Proof:** See A.5 in S1 Appendix.

Proposition 9 analyzes the magnitude of the PBS’s expected profit under different scenarios. Specifically, conclusion ① indicates that compared with decentralized decision-making, both the CS and RS contracts can increase the PBS’s expected profit [54,57]. The reason is that under both decentralized and CS decisions, the PBS’s sales profit (w−c)Q is equal. Notably, the CS contract stimulates the PBS to increase R&D investment, which increases the success probability and reduces outcome uncertainty. Therefore, the PBS’s expected revenue increases. Additionally, under the CS contract, the NEVM shares part of the R&D cost, directly reducing the PBS’s effective R&D cost, creating a cost-saving effect that increases the PBS’s expected profit. Therefore, $\pi_{\text{2}}^{CS*}>\pi_{\text{2}}^{DD*}$. Under the RS contract, the PBS’s unit profit equals that under decentralized decision-making. Although R&D cost is higher under the RS contract, market demand and success probability are also higher, so $\pi_{\text{2}}^{RS*}>\pi_{\text{2}}^{DD*}$.

Conclusion ② indicates that the effectiveness of CS versus RS contracts in improving the PBS’s profit depends on the combined effects of *t* and *s*. Differing from Zheng et al. [57], we find that when *t* is large or *s* is small, the CS contract is more effective. The reason is that a large *t* means the NEVM bears more R&D cost, directly reducing the PBS’s cost, leading to higher expected profit. However, if *s* is large or *t* is small, the RS contract is superior. The reason is that an increase in s has two effects: it directly increases the PBS’s expected sales profit, and it increases market sales. Under these combined effects, the PBS’s expected profit is larger.

**Proposition 10:**

①$\pi_{T}^{CD*}>\pi_{T}^{CS*},\;\pi_{T}^{CD*}>\pi_{T}^{RS*}>\pi_{T}^{DD*}.$
②$\left\{ \begin{array}{c} \pi_{T}^{DD*}>\pi_{T}^{CS*}\;,\;if\;\text{3}t/\text{2}+ln(\text{1}-t)<\text{0}\\ \pi_{T}^{DD*}<\;\pi_{T}^{CS*},\;if\;\text{3}t/\text{2}+ln(\text{1}-t)>\text{0} \end{array}\right.\;.$
③$\left\{ \begin{array}{c} \pi_{T}^{CS*}>\pi_{T}^{RS*},\;if\;\frac{s(\text{3}s-\text{4})H^{\text{2}}}{\text{1}\text{6}\alpha (\text{2}-s{)}^{\text{2}}}+\frac{\left[\text{3}t(\text{2}-s)-s\right]K}{\text{2}(\text{2}-s)}+K\;ln\frac{\text{2}(\text{1}-t)}{\left(\text{2}-s\right)}>\text{0}\\ \pi_{T}^{CS*}<\pi_{T}^{RS*},\;if\;\frac{s(\text{3}s-\text{4})H^{\text{2}}}{\text{1}\text{6}\alpha (\text{2}-s{)}^{\text{2}}}+\frac{\left[\text{3}t(\text{2}-s)-s\right]K}{\text{2}(\text{2}-s)}+K\;ln\frac{\text{2}(\text{1}-t)}{\left(\text{2}-s\right)}<\text{0} \end{array}\right.\;.$

**Proof:** The proof method is similar to Proposition 9, thus omitted.

Proposition 10 analyzes the magnitude of the supply chain’s expected profit under different scenarios. Conclusion ① indicates that decentralized decision-making, CS, and RS contracts cannot achieve the supply chain expected profit under centralized decision-making. This differs from the conclusions of Xu [50] and Cai et al. [53]. Compared to decentralized and RS decisions, although R&D investment is larger under centralized decision-making, supply chain sales profit (p−c)Qand success probability are also larger. Therefore, $\pi_{T}^{DD*}$ and $\pi_{T}^{RS*}$ are both less than $\pi_{T}^{CD*}$. Under the CS contract, when 0 < *t* < 0.5, although R&D investment is larger under centralized decision-making, the success probability is also larger. At this point, the higher expected profit from the higher probability exceeds that under the CS contract. When 0.5<t<1, R&D investment under centralized decision-making is lower than that under the CS contract, leading to a lower R&D success probability. Nevertheless, the centralized decision-making benchmark still generates a higher expected supply chain profit than the CS contract because the reduction in R&D cost and the increase in expected operating profit jointly outweigh the loss caused by the lower success probability. Furthermore, conclusion ① also indicates that compared to decentralized decision-making, the RS contract can increase the expected profit of the supply chain [11,57]. Compared to decentralized decision-making, although R&D cost is larger under the RS contract, sales profit (p−c)Q and success probability are also larger, leading to higher supply chain expected profit.

Conclusion ② indicates that, compared with decentralized decision-making, the CS contract does not necessarily increase the expected profit of the supply chain. This result is consistent with Wang et al. [55]. Notably, under both decentralized and CS decisions, the supply chain’s sales profit (p−c)Q is equal. Under decentralized decision-making, R&D cost is lower than under the CS contract. However, the success probability is higher under the CS contract. When t is small, under the CS contract, the PBS bears higher R&D cost, exceeding the additional expected sales profit from the higher success rate, so $\pi_{T}^{DD*}>\pi_{T}^{CS*}$. When *t* is large, under the CS contract, the PBS bears lower R&D cost, less than the additional expected sales profit, so $\pi_{T}^{DD*}<\;\pi_{T}^{CS*}$.

Conclusion ③ indicates that the CS contract is not necessarily superior to the RS contract in improving the supply chain’s expected profit. However, differing from Wang [11] and Zheng et al. [57], we find that the CS contract is superior only when *t* is relatively large or *s* is extremely small. The reason is that, as mentioned, sales profit (p−c)Q is larger under the RS contract compared to the CS contract. However, an increase in t leads to increased R&D cost and success rate. Therefore, the CS contract is superior only under specific conditions.

## 5. Numerical simulations

This section uses numerical analysis to primarily examine: (1) the impact of R&D capability on equilibrium solutions; (2) the impact of R&D subsidies on equilibrium solutions; (3) a comparative analysis of different contracts’ effects. The initial parameter settings are: *A* = 12, *β* = 0.8, *α* = 1, *c* = 0.5, $\Delta_{b}$= 2, *t* = 0.2, *s* = 0.1, *η* = 0.2, *λ* = 1.2.

To verify that the main conclusions are not driven by a single parameter set, we repeat the numerical analysis under alternative parameter combinations. The results are reported in S2 Appendix, and the qualitative conclusions remain unchanged.

### 5.1 Impact of R&D Capability on R&D Investment and Expected Profits

Fig 1 illustrates the impact of R&D capability on investment. Figs 1(a) and 1(b) visually demonstrate the inverted U-shaped relationship between R&D capability and investment. When capability is low, investment increases with capability. When capability reaches a threshold ($\lambda^{N*}$), further enhancement leads to decreased investment. This aligns with conclusion ① of Proposition 5. Furthermore, a comparison of Figs 1(a) and 1(b) shows that $I^{DD*}$ is the smallest. Fig 1(b) shows that the relative magnitude of R&D investment under centralized decision-making versus the CS contract depends on the CS ratio *t*. When the NEVM bears a larger share (0.5 < *t* < 1), $I^{CD*}<I^{CS*}$. When the NEVM bears a smaller share (0 < *t* < 0.5), $I^{CD*}>I^{CS*}$. This verifies the correctness of conclusions ①② in Proposition 7.

**Fig 1:**
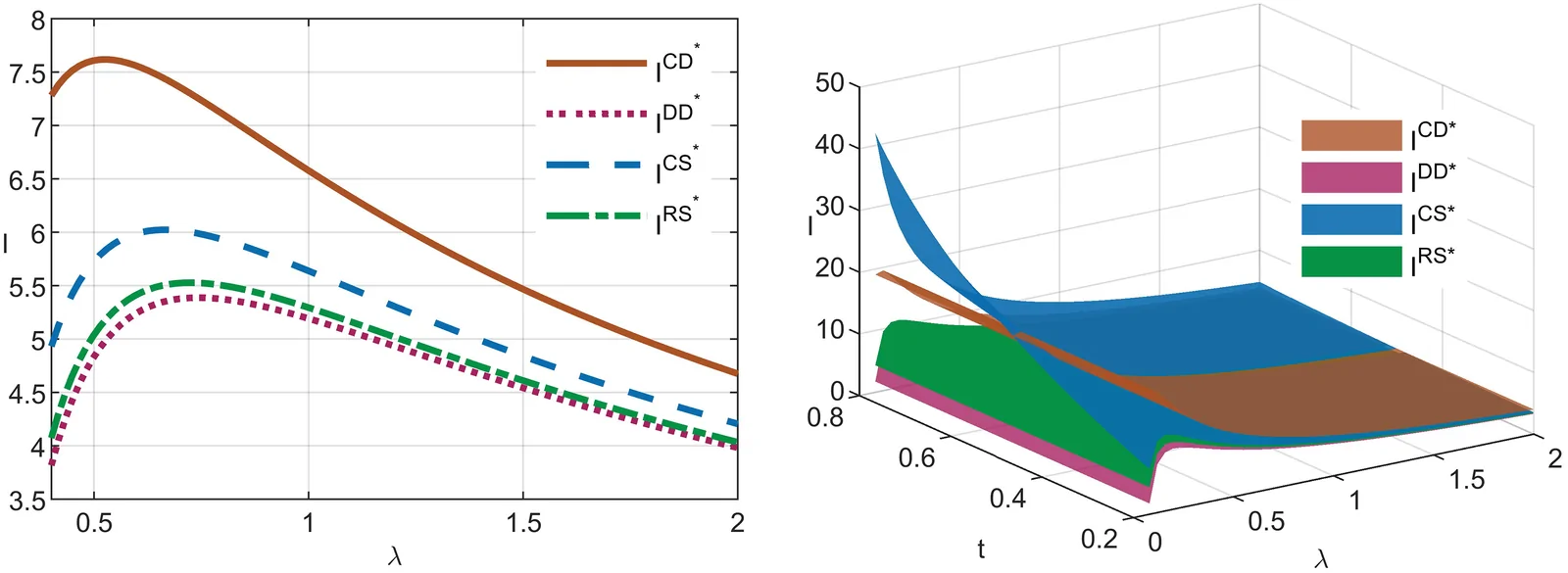
Impact of R&D capability on investment. a. Impact of λ on $I^{N*}$ b. Impact of λ and *t* on $I^{N*}$.

Fig 2 illustrates the impact of R&D capability on expected profits. Fig 2(a) shows that under decentralized decision-making and the RS contract, the NEVM’s expected profit increases with R&D capability. Additionally, Fig 2(a) shows that the plots of $\pi_{\text{1}}^{DD*}$ intersect with those of $\pi_{\text{1}}^{CS*}$ and $\pi_{\text{1}}^{RS*}$, indicating that CS and RS contracts can only improve the NEVM’s expected profit under certain conditions, further verifying conclusions ①② of Proposition 8. Fig 2(b) visually demonstrates the U-shaped relationship between the NEVM’s expected profit and R&D capability under the CS contract, meaning that under certain conditions, profit increases as capability increases. Figs 2(c) and 2(d) show that the expected profits of the PBS and the supply chain increase with enhanced R&D capability. The results shown in these four figures align with conclusions ②-⑤ of Proposition 5. Notably, Fig 2(d) also shows that the plots of $\pi_{T}^{CS*}$ and $\pi_{T}^{RS*}$ intersect, indicating that the CS contract is not always superior to the RS contract.

**Fig 2:**
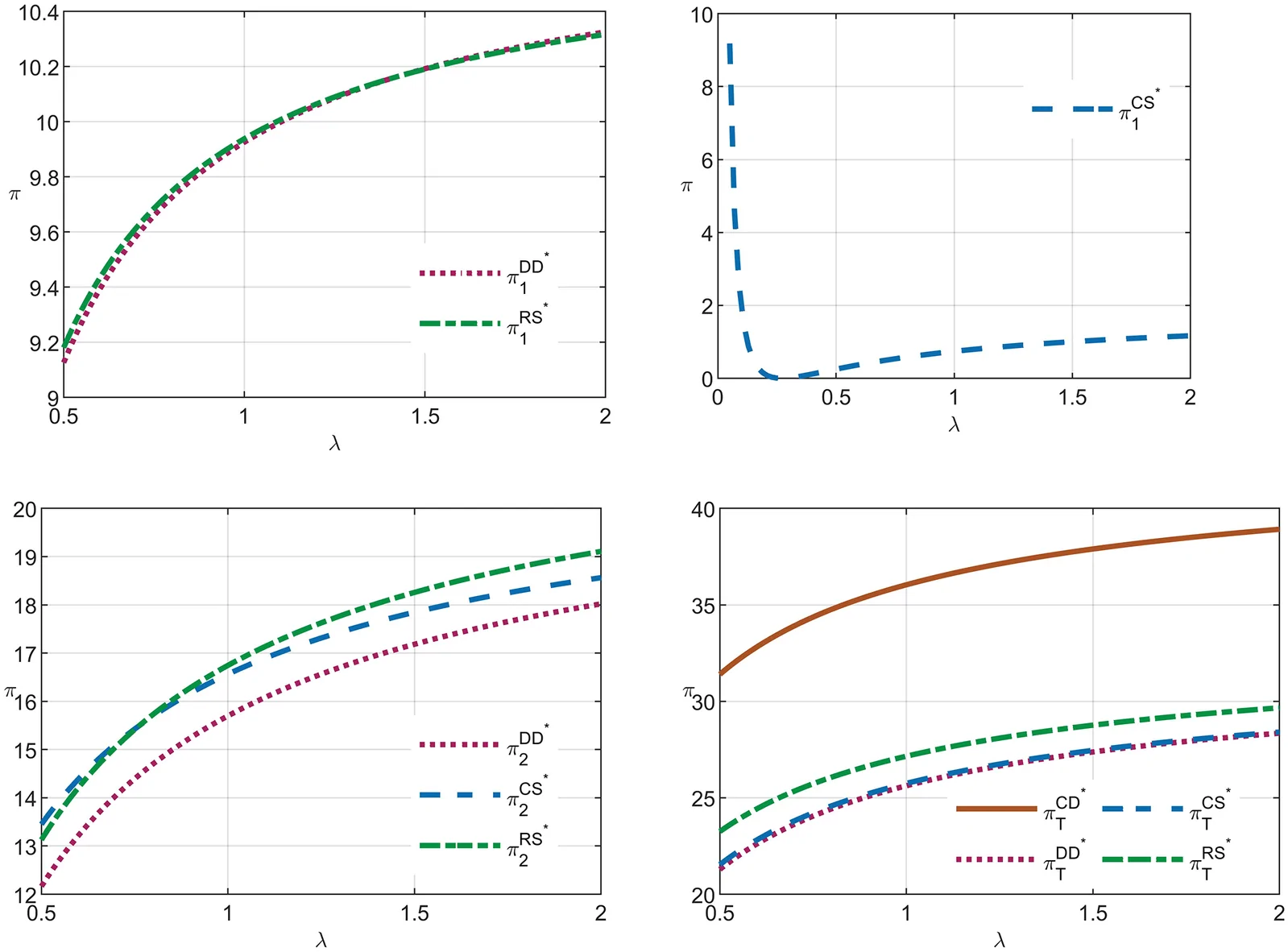
Impact of R&D capability on expected profits. a. Impact of λ on $\pi_{\text{1}}^{DD*}$ and $\pi_{\text{1}}^{RS*}$ b. Impact of λ on$\pi_{\text{1}}^{CS*}$. c. Impact of λ on $\pi_{\text{2}}^{DD*}$, $\pi_{\text{2}}^{CS*}$ and $\pi_{\text{2}}^{RS*}$ d. Impact of λ on$\pi_{T}^{N*}$.

### 5.2 Impact of R&D Subsidies on R&D Investment and Expected Profits

Fig 3 illustrates the impact of R&D subsidies on investment. Figs 3(a) and 3(b) show that government R&D subsidies have a significant incentive effect: as subsidies increase, PBS R&D investment increases. This aligns with conclusion ① of Proposition 6. Furthermore, a comparison of Figs 3(a) and 3(b) shows that $I^{DD*}$ is the smallest. Similar to Fig 1(b), Fig 3(b) visually shows that the relative magnitude of R&D investment under centralized decision-making versus under the CS contract depends on *t*. When the NEVM bears a small share (0 < *t* < 0.5), $I^{CD*}>I^{CS*}$. When the NEVM bears a large share (0.5 < *t* < 1), $I^{CD*}<I^{CS*}$. Specifically, Fig 3(c) visually shows that the relative magnitude of R&D investment under the two contracts depends on *t* and *s*. This also confirms Proposition 7.

**Fig 3:**
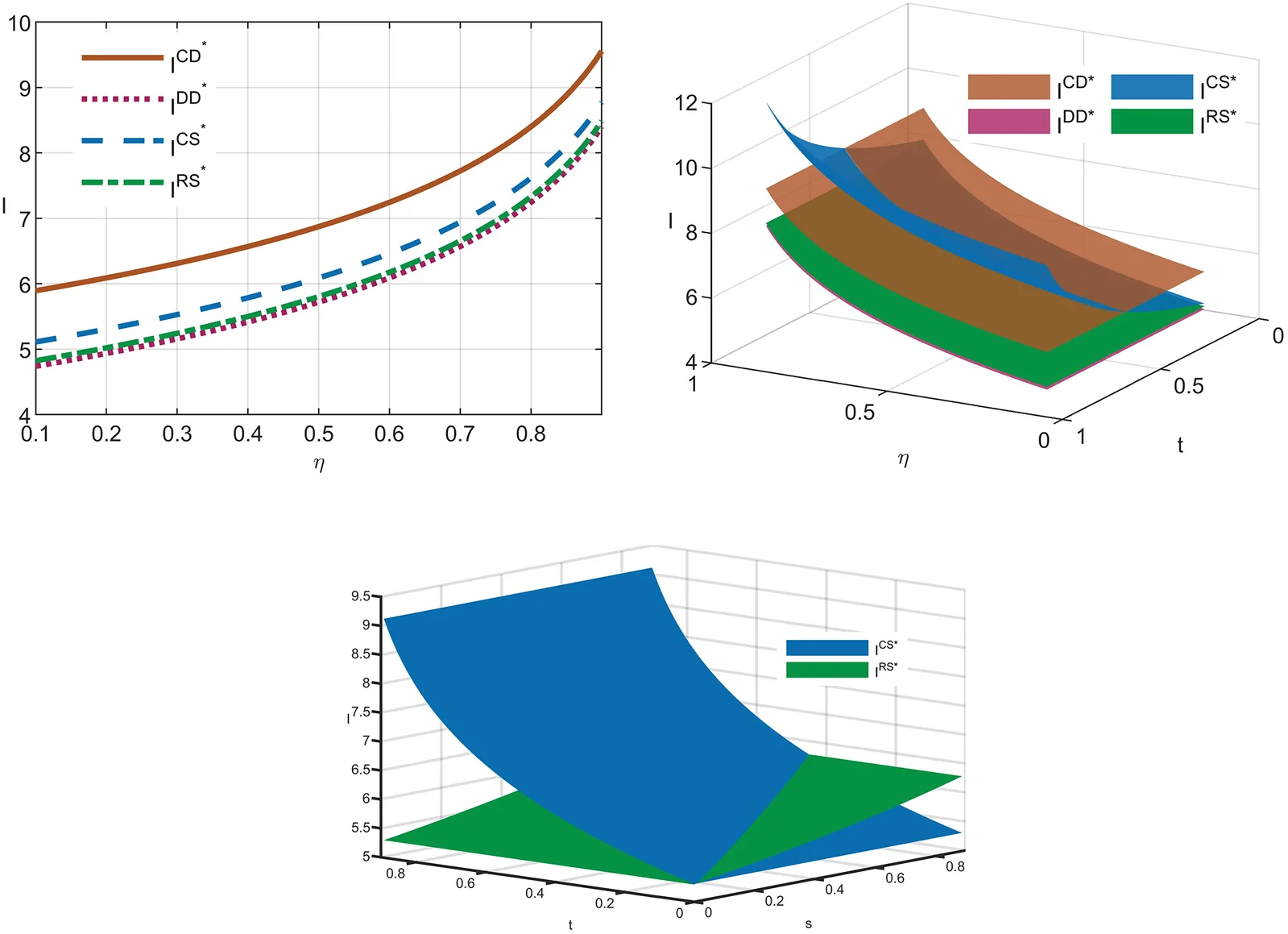
Impact of R&D subsidies on investment. a. Impact of η on $I^{N*}$ b. Impact of η and t on$I^{N*}$c. Comparison between $I^{CS*}$ and$I^{RS*}$.

Fig 4 illustrates the impact of R&D subsidies on expected profits. According to Fig 4(a), under decentralized decision-making and the RS contract, the NEVM’s expected profit increases as higher subsidies. Additionally, Fig 4(a) shows that the plots of $\pi_{\text{1}}^{DD*}$ and $\pi_{\text{1}}^{RS*}$ intersect, meaning the RS contract can only improve the NEVM’s expected profit under certain conditions, further verifying conclusion ① of Proposition 8. Fig 4(b) shows that under the CS contract, there is a U-shaped relationship between the NEVM’s expected profit and subsidies. Figs 4(c) and 4(d) show that the expected profits of the PBS and the supply chain increase with higher subsidies. The results shown in these four figures align with conclusions ②-⑤ of Proposition 6.

**Fig 4:**
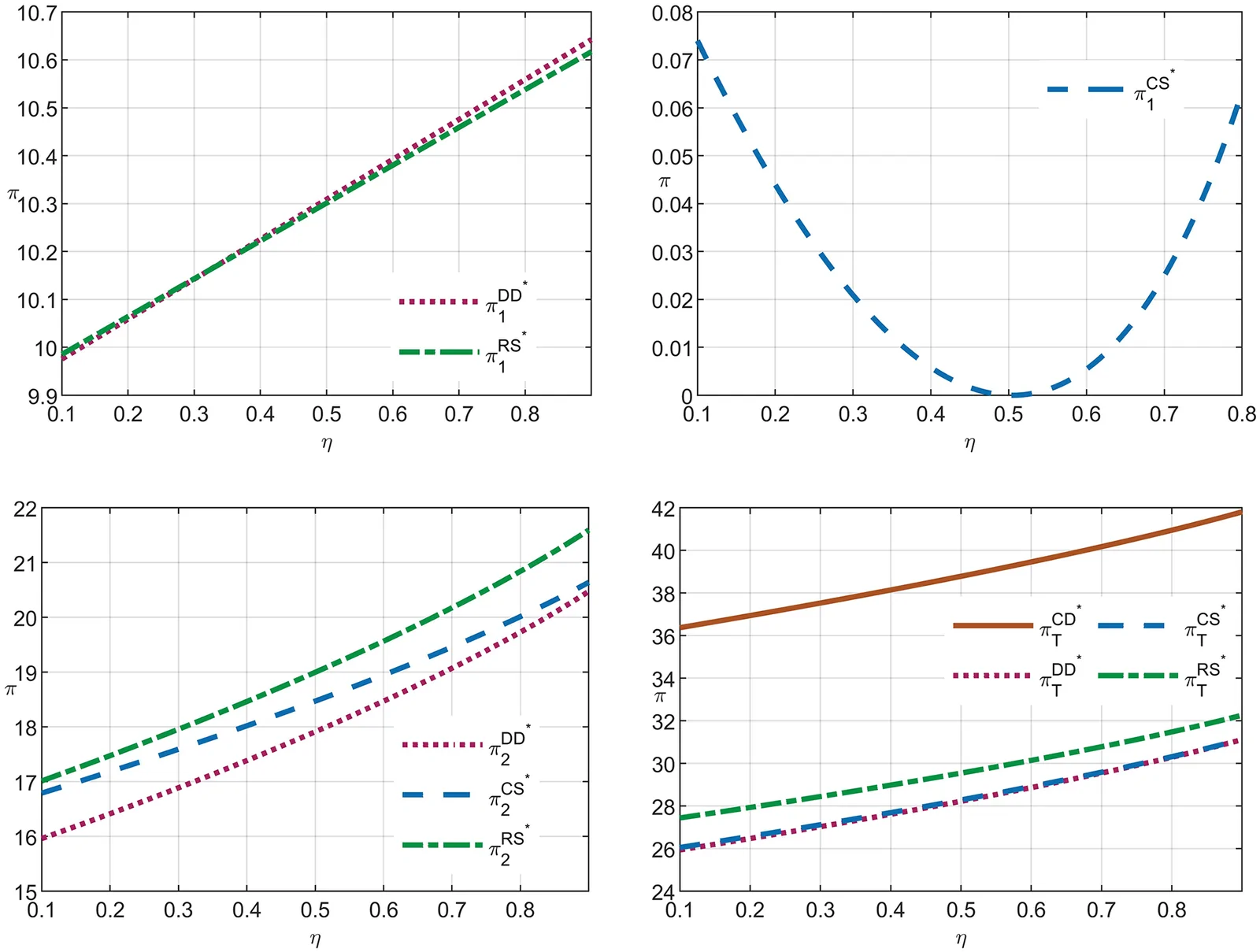
Impact of R&D subsidies on expected profits. a. Impact of *η* on $\pi_{\text{1}}^{DD*}$ and $\pi_{\text{1}}^{RS*}$ b. Impact of *η* on$\pi_{\text{1}}^{CS*}$c. Impact of *η* on $\pi_{\text{2}}^{DD*},\;\pi_{\text{2}}^{CS*}\text{ and }\pi_{\text{2}}^{RS*}$ d. Impact of *η* on$\pi_{T}^{N*}$.

### 5.3 Comparative Analysis of Different Contracts

Fig 5 illustrates the impact of different contracts on the NEVM’s expected profit. Figs 5(a) and 5(b) show that, compared with decentralized decision-making, CS and RS contracts do not necessarily improve the NEVM’s expected profit. In other words, such improvement occurs only under certain conditions. Furthermore, Fig 5(c) shows that the relative effectiveness of the two contracts depends on the combined effects of *t* and *s*. This confirms Proposition 8.

**Fig 5:**
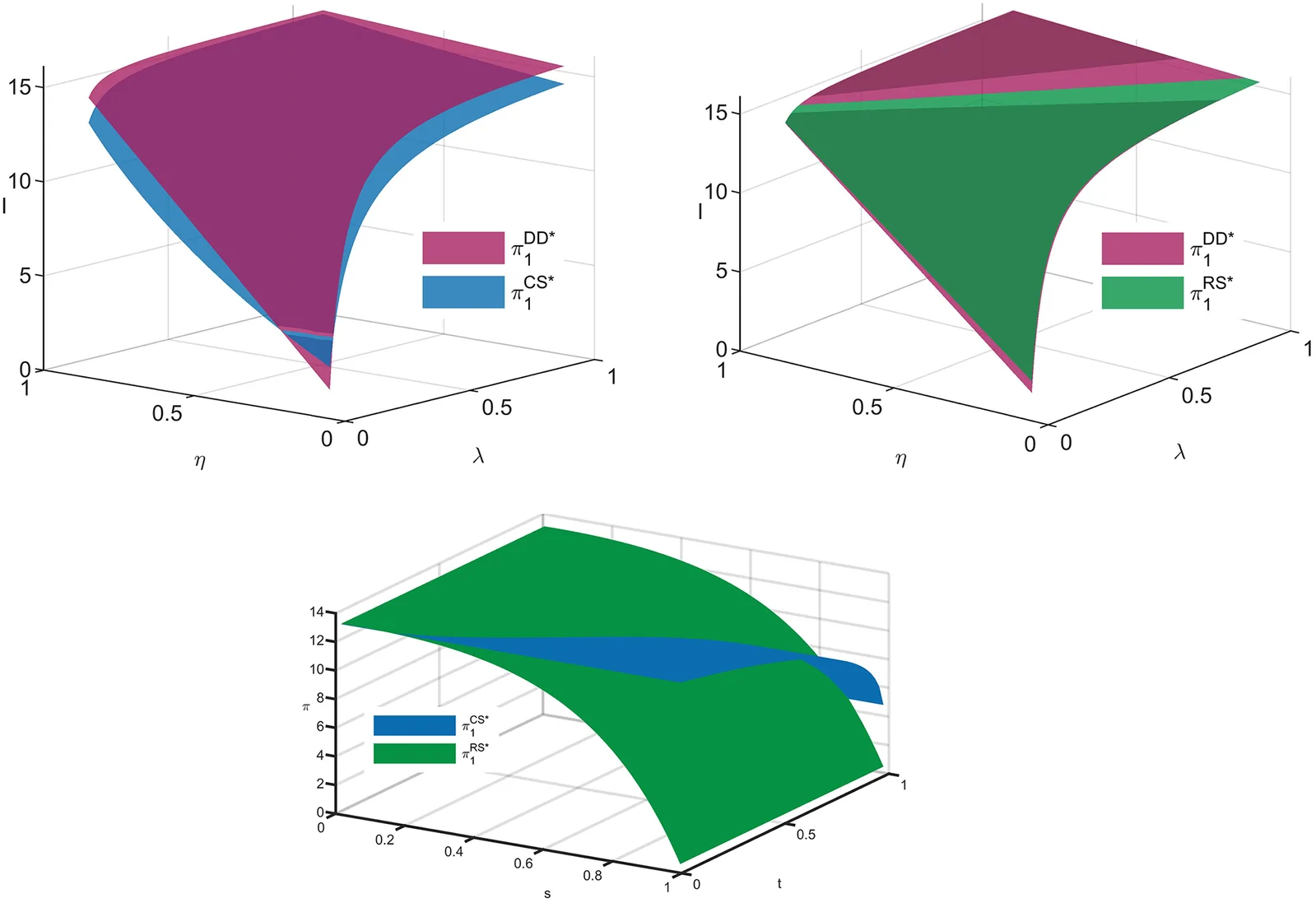
Impact of different contracts on the NEVM’s expected profit. a. Comparison between $\pi_{\text{1}}^{DD*}$and $\pi_{\text{1}}^{CS*}$ b. Comparison between $\pi_{\text{1}}^{DD*}$and$\pi_{\text{1}}^{RS*}$. c. Comparison between $\pi_{\text{1}}^{CS*}$ and $\pi_{\text{1}}^{RS*}$.

Fig 6 illustrates the impact of different contracts on the PBS’s expected profit. Fig 6(a) shows that, compared to decentralized decision-making, both CS and RS contracts can increase the PBS’s expected profit. As discussed, under these contracts, the NEVM directly or indirectly compensates for the PBS’s R&D cost, generating three effects: direct effects, indirect effects, and cost compensation effects. Under these combined effects, the PBS’s expected profit under decentralized decision-making is lower than under the two contracts. Furthermore, Fig 6(b) shows the relative effectiveness of the two contracts in improving the PBS’s profit. The CS contract’s superiority is apparent only when *t* is large and *s* is small. This verifies Proposition 9.

**Fig 6:**
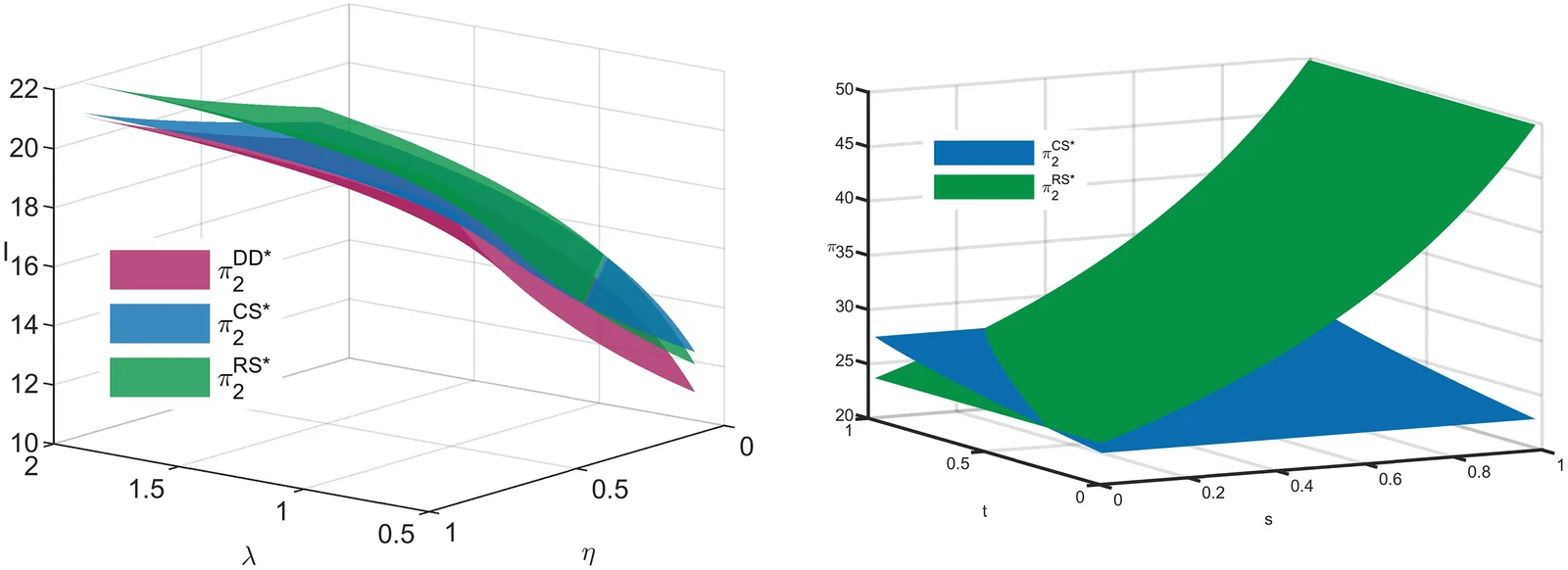
Impact of different contracts on the PBS’s expected profit. a. Comparison among $\pi_{\text{2}}^{DD*},\;\pi_{\text{2}}^{CS*}\text{ and }\pi_{\text{2}}^{RS*}$ b. Comparison among $\pi_{\text{2}}^{DD*},\;\pi_{\text{2}}^{CS*}\text{ and }\pi_{\text{2}}^{RS*}$.

Fig 7 illustrates the impact of different contracts on the supply chain’s expected profit. According to Figs 7(a) and 7(b), the supply chain’s expected profit is maximized under centralized decision-making. That is, decentralized, CS, and RS decisions cannot achieve optimal supply chain expected profit. We also find that the relative magnitude of supply chain’s expected profit under decentralized decision-making versus under the CS contract depends on t. When *t* is small and the condition 3t/2+ln(1−t)<0 is satisfied, the supply chain’s expected profit is larger under decentralized decision-making than under the CS contract. When *t* is large and the condition 3t/2+ln(1−t)>0is satisfied, the supply chain’s expected profit is larger under the CS contract than under decentralized decision-making. Figs 7(a) and 7(b) show that, compared with the decentralized case, the RS contract can improve the supply chain’s expected profit, thereby achieving supply chain coordination. Additionally, Fig 7(c) shows that only under specific conditions is the CS contract superior to the RS contract in improving supply chain expected profit. This verifies the correctness of Proposition 10.

**Fig 7:**
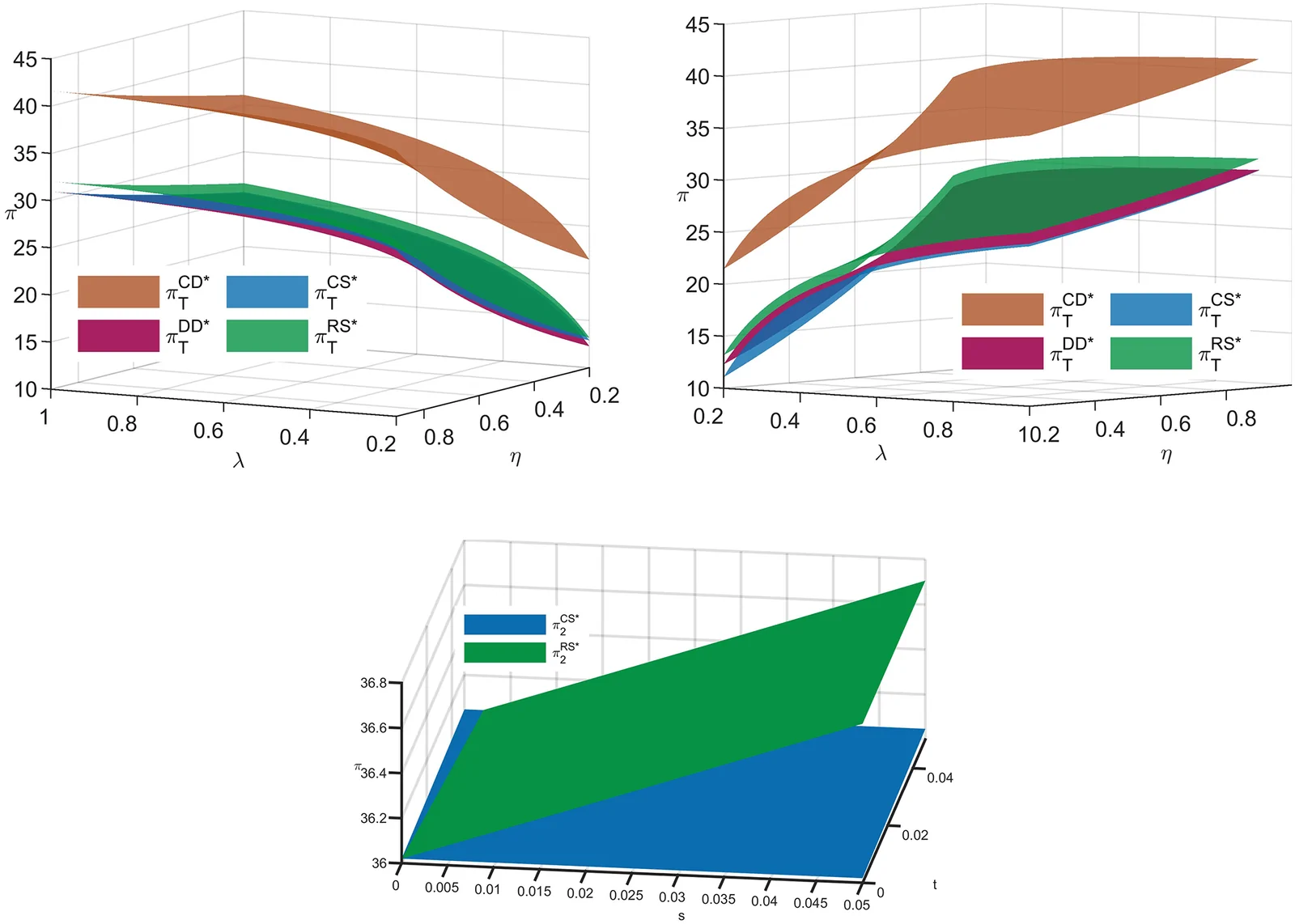
Impact of different contracts on the supply chain’s expected profit. a. $\begin{array}{c} \pi_{T}^{CD*},\;\pi_{T}^{DD*},\;\pi_{T}^{CS*}\text{and }\pi_{T}^{RS*}\;(t<0.5) \end{array}$ b.$\begin{array}{c} \pi_{T}^{CD*},\;\pi_{T}^{DD*},\;\pi_{T}^{CS*}\text{ and }\pi_{T}^{RS*}\;(t>0.5) \end{array}$. c. Comparison between $\pi_{T}^{CS*}\text{ and }\pi_{T}^{RS*}$.

## 6. Discussion

Based on the theoretical analysis, comparative analysis, and numerical simulation results, this section summarizes the corresponding theoretical insights and derives managerial implications for the government, the NEVM, and the PBS. Note that the following implications should be interpreted as managerial extensions of the model rather than as direct predictions regarding unmodeled institutional or organizational mechanisms.

First, for the government, it is essential to fully account for heterogeneity in firms’ R&D capabilities and cooperation modes and to carefully evaluate the effectiveness of R&D subsidies. Changes in the R&D subsidy rate affect firms’ R&D investment and the expected profits of supply chain members; however, the specific effects depend on the level of R&D capability and the contractual arrangement. Moreover, the inverted U-shaped relationship between R&D capability and optimal R&D investment indicates that R&D investment does not increase monotonically with R&D capability. Therefore, when designing and evaluating R&D support measures, the government should not simply equate a higher subsidy rate with improved R&D performance. Instead, it should take into account firms’ technological capabilities, the characteristics of R&D projects, and cooperative relationships to assess the actual effects of subsidies on R&D investment and expected profits. For example, for a PBS in the capability ramp-up phase ($\lambda <\lambda^{N^{*}}$), policy support could focus on alleviating the upfront investment burden of R&D activities, which suggests that the government should increase R&D subsidies. In particular, power batteries currently face technical bottlenecks in core performance dimensions such as battery modularization and driving range. Government subsidies should therefore be directed toward key component firms with the most urgent innovation needs, encouraging PBSs to increase R&D investment, overcome core technical bottlenecks, and alleviate consumer “range anxiety.” By contrast, for a PBS in the mature-capability phase ($\lambda >\lambda^{N^{*}}$), to prevent excessive R&D investment, the government should gradually reduce direct subsidies and place greater emphasis on the efficiency of R&D funding and the actual output of technological achievements. In addition, measures such as R&D risk compensation funds, tax incentives, and industry–university–research collaboration platforms may help mitigate R&D uncertainty and promote technological synergy. It should be noted that this paper treats the government’s R&D subsidy rate as an exogenous parameter and does not formulate an optimization model of government subsidy decisions. Accordingly, the above policy recommendations are derived mainly from the comparative analysis of the model, while suggestions concerning specific policy instruments represent practical extensions of the research findings.

Second, NEVMs should adopt differentiated cooperative contracts based on the R&D capability of the PBS. On one hand, they should actively seek cooperation with PBSs possessing high R&D capability. High capability implies a higher probability of R&D success. Leading scientific teams, advanced experimental facilities, and extensive R&D experience can facilitate fundamental breakthroughs at the chemical level (e.g., in solid-state electrolytes, cathode/anode materials). Concurrently, NEVMs need to form dedicated teams to conduct comprehensive assessments of PBSs’ R&D capabilities from multiple dimensions—including technical depth, innovation efficiency, practical application potential, and intellectual property—to accurately evaluate capability and mitigate potential “moral hazard.” On the other hand, for PBSs with sufficiently high R&D capability, NEVMs may have less need to rely on cost-sharing mechanisms, because such PBSs can achieve a relatively high probability of R&D success with lower R&D investment. Therefore, a decentralized decision-making structure may be suitable. High intrinsic capability often implies sufficient internal investment resources and relatively low perceived R&D risk, allowing the NEVM to secure high-performance batteries without sharing R&D costs. For PBSs with moderate capability, CS or RS contracts are advisable. When a PBS has medium capability and is prone to underinvestment, the NEVM’s contribution to a portion of the R&D cost can alleviate the PBS’s financial constraints and significantly boost its R&D investment. Both parties should negotiate reasonable CS and RS ratios to avoid excessive investment.

Finally, for PBSs, on one hand, they should focus R&D efforts on breakthrough technologies and proactively leverage government R&D subsidy policies to reduce innovation risk. On the other hand, they must recognize the inverted U-shaped relationship between R&D capability and investment to avoid investment traps. For PBSs in the ramp-up phase (λ <$\lambda^{\text{N}*}$), sustained and increased resource investment is necessary to enhance the probability of R&D success, reduce outcome uncertainty, expand market share, and build technological advantages. For PBSs in the mature phase (λ> $\lambda^{\text{N}*}$), after reaching a high capability level, merely pursuing further improvements in capability metrics may yield diminishing returns. Therefore, PBSs should reduce marginal R&D investment to avoid the overinvestment trap and shift their strategy toward efficiency and cost optimization, technology licensing, and knowledge monetization to secure higher returns. Additionally, when cooperating with NEVMs, PBSs should actively seek CS or RS contracts to incentivize the NEVM to directly or indirectly share R&D costs, thereby mitigating their own R&D risk. Notably, the RS contract can simultaneously improve both the PBS’s and the overall supply chain’s expected profits. Both parties should engage in negotiations to determine mutually beneficial CS and RS ratios to achieve a win-win outcome.

## 7. Conclusion

This paper examines a supply chain consisting of a single NEVM and a single PBS. Considering government subsidies and R&D outcome uncertainty, it first employs an exponential function to characterize R&D outcome uncertainty based on the combined effects of R&D difficulty, R&D capability, and R&D investment. Subsequently, it designs CS and RS contracts, with a focus on analyzing the impacts of R&D subsidies and R&D capability on R&D investment, the supply chain, and its members’ expected profits. Finally, a comparative analysis of the performance of different contractual arrangements is conducted.

The main findings are as follows: (1) R&D investment exhibits an inverted U-shaped relationship with R&D capability and a positive relationship with R&D subsidies. (2) Under the RS contract, the expected profits of the supply chain and its members increase with higher R&D subsidies and enhanced R&D capability. Under the CS contract, the expected profits of the PBS and the supply chain increase with higher R&D subsidies and enhanced R&D capability; however, the NEVM’s expected profit demonstrates a U-shaped relationship with both R&D subsidies and R&D capability. (3) Compared to the centralized decision-making benchmark, neither contract achieves the centralized benchmark for the supply chain’s expected profit. However, compared to decentralized decision-making, the CS contract increases R&D investment and the PBS’s expected profit; the RS contract simultaneously increases R&D investment, the PBS’s expected profit, and the supply chain’s expected profit. Both contracts can improve the NEVM’s expected profit only under certain conditions. Furthermore, the relative superiority of the CS contract versus the RS contract depends on the combined effects of the CS ratio and the RS ratio.

While this study examines cooperative R&D contract design for power batteries under R&D subsidies and outcome uncertainty, it has certain limitations. (1) The model focuses on a supply chain with a single NEVM and a single PBS, neglecting competitive interactions among multiple firms. Future research could extend the analysis to settings with multiple NEVMs or multiple PBSs, incorporating competitive dynamics into the model. (2) Although this study compares the performance of CS and RS contracts, it does not derive the optimal CS and RS ratios. Determining these optimal parameters represents a valuable direction for future work.

## Supporting information

S1 Appendix Proof of propositions. (DOCX)

S2 Appendix Robustness results of numerical analysis. (DOCX)

S1 Program Matlab program for Figs.1–7. (DOCX)
