# Supplementary material for: Cooperative R&D contracts for power batteries under R&D outcome uncertainty and subsidies
Source: PLoS One. 2026 Nov 11;21(11):e0362349. doi: 10.1371/journal.pone.0362349 (PMC13754133; doi:10.1371/journal.pone.0362349)
Supplement: Supplementary file 1 [file pone.0362349.s001.docx]

# Appendix

## A.1: Proof of Proposition 1

**Proof:**

Under the DD, the expected profits of the NEVM and the PBS are given by:

$\pi_{1}^{DD}=[1-exp(-\lambda I/\Delta_{b})]\left( p-w \right)\left( A-\alpha p+\beta\Delta_{b} \right)$ (A.1)

$\pi_{2}^{DD}=[1-exp(-\lambda I/\Delta_{b})]\left( w-c \right)\left( A-\alpha p+\beta\Delta_{b} \right)-(1-\eta)I$ (A.2)

Differentiating Equation (A.1) with respect to p and setting $\partial\pi_{1}^{DD}/\partial p=0$ yields:

$p=(A+\beta\Delta_{b}+\alpha w)/2\alpha$ (A.3)

Substituting (A.3) into (A.2) gives:

$\pi_{2}^{DD}=[1-exp(-\lambda I/\Delta_{b})](w-c) (A+\beta\Delta_{b}-\alpha w)/2-(1-\eta)I$ (A.4)

Solving the first-order conditions $\partial\pi_{2}^{DD}/\partial w=0$ and $\partial\pi_{2}^{DD}/\partial I=0$ simultaneously from (A.6):

$w^{DD*}=(A+\beta\Delta_{b}+\alpha c)/2\alpha$*,* $I^{DD*}=\Delta_{b} ln [\lambda\left( A+\beta\Delta_{b}-\alpha c \right)^{2}/8\alpha(1-\eta)\Delta_{b}]/\lambda$ (A.5)

The Hessian matrix $E^{WP}$ of $\pi_{2}^{WP}$ is:

$E^{DD}=\left[ \begin{matrix} -\alpha[1-exp(-\lambda I/\Delta_{b})] & \lambda exp(-\lambda I/\Delta_{b})\left[ (A+\beta\Delta_{b}-\alpha w)-\alpha(w-c) \right]/{2\Delta}_{b} \\ \lambda exp(-\lambda I/\Delta_{b})\left[ (A+\beta\Delta_{b}-\alpha w)-\alpha(w-c) \right]/{2\Delta}_{b} & -\lambda^{2}exp(-\lambda I/\Delta_{b})\left( w-c \right)(A+\beta\Delta_{b}-\alpha w)/2{\Delta_{b}}^{2} \end{matrix} \right]$(A.6)

Since First-order leading principal minor *D₁ < 0* and second-order leading principal minor *D₂ > 0*, the Hessian matrix $E^{DD}(w,I)$ is negative definite. This confirms that $w^{DD*}$ and $I^{DD*}$ constitute the unique globally optimal solution to the decision problem.  Consequently, we derive: $p^{DD*}=\left[ 3\left( A+\beta\Delta_{b} \right)+\alpha c \right]/4\alpha$*,*$Q^{DD*}=(A+\beta\Delta_{b}-\alpha c)/4$*,*$\pi_{1}^{DD*}=\left( A+\beta\Delta_{b}-\alpha c \right)^{2}/16\alpha-\left( 1-\eta\right)\Delta_{b}/2\lambda$*,*$\pi_{2}^{DD*}=\left( A+\beta\Delta_{b}-\alpha c \right)^{2}/8\alpha-\left( 1-\eta\right)\Delta_{b}/\lambda\{1+ ln[\lambda\left（ A+\beta\Delta_{b}-\alpha c \right）^{2}/8\alpha(1-\eta)\Delta_{b}]\}$*,*$\pi_{T}^{DD*}=3\left( A+\beta\Delta_{b}-\alpha c \right)^{2}/16\alpha-\left( 1-\eta\right)\Delta_{b}/\lambda\{\frac{3}{2}+ ln[\lambda\left（ A+\beta\Delta_{b}-\alpha c \right）^{2}/8\alpha(1-\eta)\Delta_{b}]\}$.

## A.2: Proof of Proposition 2

**Proof:**

Under the CD, the expected profit of the supply chain $\pi_{T}^{CD}$ is expressed as:

$\pi_{T}^{CD}=[1-exp(-\lambda I/\Delta_{b})]\left( p-c \right)\left( A-\alpha p+\beta\Delta_{b} \right)-(1-\eta)I$ (A.7)

From Equation (A.1), we solve the system of first-order conditions $\partial\pi_{T}^{CD}/\partial p=0$ and $\partial\pi_{T}^{CD}/\partial I=0$ to obtain the optimal solutions:

$p^{CD*}=(A+\beta\Delta_{b}+\alpha c)/2\alpha,I^{CD*}=\Delta_{b} ln[{\lambda\left（ A+\beta\Delta_{b}-\alpha c \right）}^{2}/4\alpha{\left( 1-\eta\right)\Delta}_{b}]/\lambda$*.*

The Hessian matrix $E^{CD}$ of $\pi_{T}^{CD}$ is given by:

$E^{CD}=\left[ \begin{matrix} -2\alpha[1-exp(-\lambda I/\Delta_{b})] & \lambda exp(-\lambda I/\Delta_{b})[\left( A-\alpha p+\beta\Delta_{b} \right)-\alpha\left( p-c \right)]/\Delta_{b} \\ \lambda exp(-\lambda I/\Delta_{b})[\left( A-\alpha p+\beta\Delta_{b} \right)-\alpha\left( p-c \right)]/\Delta_{b} & -{(\lambda/\Delta_{b})}^{2}exp(-\lambda I/\Delta_{b})\left( p-c \right)\left( A-\alpha p+\beta\Delta_{b} \right) \end{matrix} \right]$(A.8)

Since First-order leading principal minor *D₁ < 0* and second-order leading principal minor *D₂ > 0*, the Hessian matrix $E^{CD}(p, I)$ is negative definite. This confirms that $p^{CD*}$ and $I^{CD*}$ constitute the unique globally optimal solution to the decision problem.  Consequently, we derive: $Q^{CD*}=(A+\beta\Delta_{b}-\alpha c)/2$,$\pi_{T}^{CD*}=\left（ A+\beta\Delta_{b}-\alpha c \right）^{2}/4\alpha-\left( 1-\eta\right)\Delta_{b}/\lambda-\left( 1-\eta\right)\Delta_{b}ln[{\lambda\left（ A+\beta\Delta_{b}-\alpha c \right）}^{2}/4\alpha(1-\eta)\Delta_{b}]/\lambda$.

## A.3: Proof of Proposition 5

**Proof:**

**(1)** Under the CD, the partial derivative of $I^{CD*}$ with respect to *λ* is given by:

$\partial I^{CD*}/\partial\lambda=\Delta_{b}\{1-ln[{\lambda\left（ A+\beta\Delta_{b}-\alpha c \right）}^{2}/4\alpha{\left( 1-\eta\right)\Delta}_{b}]\}/\lambda^{2}$*.*

Setting $\partial I^{CD*}/\partial\lambda=0$ yields $\lambda^{CD*}=4e\alpha{(1-\eta)\Delta}_{b}/\left（ A+\beta\Delta_{b}-\alpha c \right）^{2}$. Hence, *if* $0<\lambda<\lambda^{CD*}$*，*then $\partial I^{CD*}/\partial\lambda>0$*; if* $\lambda\geq\lambda^{CD*}$*，*then $\partial I^{CD*}/\partial\lambda\leq0$.

The same proof procedure applies to the DD, CS, and RS contracts.

**(2)** Under the DD and RS, the partial derivatives of $\pi_{1}^{DD*}$ and $\pi_{1}^{RS*}$ with respect to *λ* are respectively given by:

$\partial\pi_{1}^{DD*}/\partial\lambda=\Delta_{b}\left( 1-\eta\right)/{2\lambda}^{2}$ and $\partial\pi_{1}^{RS*}/\partial\lambda=\left( 1-s \right)\left( 1-\eta\right)\Delta_{b}/\lambda^{2}\left( 2-s \right)$.

Thus, $\partial\pi_{1}^{DD*}/\partial\lambda>0$*,* $\partial\pi_{1}^{RS*}/\partial\lambda>0$.

**(3)** Under the CS, the partial derivatives of $\pi_{1}^{CS*}$ with respect to *λ* is given by:

$\partial\pi_{1}^{CS*}/\partial\lambda=\Delta_{b}\left( 1-\eta\right)\{(1-3t)/2+tln[{\lambda\left（ A+\beta\Delta_{b}-\alpha c \right）}^{2}/8\alpha(1-t){\left( 1-\eta\right)\Delta}_{b}]\}/\lambda^{2}$.

Thus, *if* $(1-3t)/2+tln[{\lambda\left（ A+\beta\Delta_{b}-\alpha c \right）}^{2}/8\alpha(1-t){\left( 1-\eta\right)\Delta}_{b}]>0, then\frac{\partial\pi_{1}^{CS*}}{\partial\lambda}>0; if (1-3t)/2+tln[{\lambda\left（ A+\beta\Delta_{b}-\alpha c \right）}^{2}/8\alpha(1-t){\left( 1-\eta\right)\Delta}_{b}]\leq0, then \partial\pi_{1}^{CS*}/\partial\lambda\leq0.$

**(4)** Under the DD, CS and RS, the partial derivatives of $\pi_{2}^{DD*}$, $\pi_{2}^{CS*}$and $\pi_{2}^{RS*}$ with respect to *λ* are respectively given by:

$$\partial\pi_{2}^{DD*}/\partial\lambda=\left( 1-\eta\right)\Delta_{b} ln[\lambda\left（ A+\beta\Delta_{b}-\alpha c \right）^{2}/8\alpha(1-\eta)\Delta_{b}]/\lambda^{2}$$

$$\partial\pi_{2}^{CS*}/\partial\lambda=\left( 1-t \right)\left( 1-\eta\right)\Delta_{b} ln[\lambda\left（ A+\beta\Delta_{b}-\alpha c \right）^{2}/8\alpha\Delta_{b}\left( 1-t \right)(1-\eta)]/\lambda^{2}$$

$$\partial\pi_{2}^{RS*}/\partial\lambda=\left( 1-\eta\right)\Delta_{b} ln[\lambda\left（ A+\beta\Delta_{b}-\alpha c \right）^{2}/4\alpha\left( 2-s \right)(1-\eta)\Delta_{b}]/\lambda^{2}$$

Thus*,* $\partial\pi_{2}^{DD*}/\partial\lambda>0$*,* $\partial\pi_{2}^{CS*}/\partial\lambda>0$*,* $\partial\pi_{2}^{RS*}/\partial\lambda>0$*.*

**(5)** Under the CD, the partial derivative of $\pi_{T}^{CD*}$ with respect to *λ* is given by:

$\partial\pi_{T}^{CD*}/\partial\lambda=\Delta_{b}\left( 1-\eta\right)ln[{\lambda\left（ A+\beta\Delta_{b}-\alpha c \right）}^{2}/4\alpha{\left( 1-\eta\right)\Delta}_{b}]/\lambda^{2}$.

Thus, $\partial\pi_{T}^{CD*}/\partial\lambda>0.$

The same proof procedure applies to the DD and RS.

However, Under the CS, the partial derivative of $\pi_{T}^{CS*}$ with respect to *λ* is given by: $\partial\pi_{T}^{CS*}/\partial\lambda=\Delta_{b}\left( 1-\eta\right)\{(1-3t)/2+ln[{\lambda\left（ A+\beta\Delta_{b}-\alpha c \right）}^{2}/8\alpha(1-t){\left( 1-\eta\right)\Delta}_{b}]\}/\lambda^{2}$.

Let $F\left( t \right)=(1-3t)/2+ln[{\lambda\left（ A+\beta\Delta_{b}-\alpha c \right）}^{2}/8\alpha(1-t){\left( 1-\eta\right)\Delta}_{b}]$. It can be mathematically derived that the minimum value of *F*(*t*) satisfies ${F\left( t \right)}_{min}=F(1/3)>0$. Thus $\partial\pi_{T}^{CS*}/\partial\lambda>0$.

## A.4: Proof of Proposition 7

**Proof:**

**(1)** Taking the difference between $I^{CD*}$ and $I^{DD*}$, we obtain:

$I^{CD*}-I^{WP*}=\Delta_{b}ln2/\lambda$.

Hence, $I^{CD*}-I^{DD*}>0$.

Similarly, we obtain:

$I^{CS*}-I^{DD*}=-\Delta_{b}\ln\left( 1-t \right)/\lambda>0$*,* $I^{RS*}-I^{DD*}=\Delta_{b}ln[2/\left( 2-s \right)]/\lambda>0$*,* $I^{CD*}-I^{CR*}=\Delta_{b}\ln\left( 2-s \right)/\lambda>0$.

Thus, $I^{CD*}>I^{DD*}$*，*$I^{CS*}>I^{DD*}$*，*$I^{CD*}>I^{RS*}>I^{DD*}$.

**(2)** Taking the difference between $I^{CD*}$ and $I^{CS*}$, we obtain:

$I^{CD*}-I^{CS*}=\Delta_{b} ln[2\left( 1-t \right)]/\lambda$.

Thus, *if* $0<t<0.5$，$\mathrm{then}I^{CD*}>I^{CR*}$；*if* $0.5\leq t<1$，then $I^{CD*}\leq I^{CR*}.$

**(3)** Taking the difference between $I^{RS*}$ and $I^{CS*}$, we obtain:

$I^{RS*}-I^{CS*}=\Delta_{b}ln[2\left( 1-t \right)/(2-s)]/\lambda$.

Thus, *if* $s<2t$*，*$\mathrm{then} I^{CS*}>I^{RS*}$；*if* $s\geq2t$，then $I^{CS*}\leq I^{RS*}.$

## A.5: Proof of Proposition 9

**Proof:**

**(1)** Taking the difference between $\pi_{2}^{DD*}$and $\pi_{2}^{CS*}$, we obtain:

$\pi_{2}^{DD*}-\pi_{2}^{CS*}=-\left( 1-\eta\right)\Delta_{b}\{t+\ln\left( 1-t \right)+t ln[\lambda\left（ A+\beta\Delta_{b}-\alpha c \right）^{2}/8\alpha\Delta_{b}\left( 1-t \right)\left( 1-\eta\right)]\}/\lambda$.

Let $F(t)=t+\ln\left( 1-t \right)+t ln[\lambda\left（ A+\beta\Delta_{b}-\alpha c \right）^{2}/8\alpha\Delta_{b}\left( 1-t \right)\left( 1-\eta\right)]$. It can be mathematically derived that the minimum value of *F*(*t*) satisfies ${F\left( t \right)}_{min}=F\left( 0 \right)=0$. Thus, $\pi_{2}^{DD*}<\pi_{2}^{CS*}$.

Similarly, we obtain:

$\pi_{2}^{RS*}-\pi_{2}^{DD*}=s\left( A+\beta\Delta_{b}-\alpha c \right)^{2}/8\alpha\left( 2-s \right)+{\left( 1-\eta\right)\Delta}_{b}ln[(2-s)/2]/\lambda$.

Since $s\left( A+\beta\Delta_{b}-\alpha c \right)^{2}/8\alpha\left( 2-s \right)\geq s\Delta_{b}\left( 1-\eta\right)/\lambda(2-s)$.

Thus, $\pi_{2}^{RS*}-\pi_{2}^{DD*}\geq\left( 1-\eta\right)\Delta_{b}\left[ s/(2-s)+ln(2-s)/2 \right]/\lambda>0$.

**(2)** Taking the difference between $\pi_{2}^{RS*}$and $\pi_{2}^{CS*}$, we obtain:

$\pi_{2}^{RS*}-\pi_{2}^{CS*}=s\left( A+\beta\Delta_{b}-\alpha c \right)^{2}/8\alpha(2-s)-\{t- ln[(2-s)/2\left( 1-t \right)]+t ln[\lambda\left（ A+\beta\Delta_{b}-\alpha c \right）^{2}/8\alpha\Delta_{b}\left( 1-t \right)\left( 1-\eta\right)]\}\left( 1-\eta\right)\Delta_{b}/\lambda$.

Thus, *if* $s\left( A+\beta\Delta_{b}-\alpha c \right)^{2}/8\alpha(2-s)-\{t- ln[(2-s)/2\left( 1-t \right)]+t ln[\lambda\left（ A+\beta\Delta_{b}-\alpha c \right）^{2}/8\alpha\Delta_{b}\left( 1-t \right)\left( 1-\eta\right)]\}\left( 1-\eta\right)\Delta_{b}/\lambda\geq0,$ then $\pi_{2}^{RS*}\leq\pi_{2}^{CS*};$ *if* $s\left( A+\beta\Delta_{b}-\alpha c \right)^{2}/8\alpha(2-s)-\{t- ln[(2-s)/2\left( 1-t \right)]+t ln[\lambda\left（ A+\beta\Delta_{b}-\alpha c \right）^{2}/8\alpha\Delta_{b}\left( 1-t \right)\left( 1-\eta\right)]\}\left( 1-\eta\right)\Delta_{b}/\lambda<0$*,* then $\pi_{2}^{RS*}>\pi_{2}^{CS*}.$
