# Supplementary material for: Cooperative R&D contracts for power batteries under R&D outcome uncertainty and subsidies
Source: PLoS One. 2026 Nov 11;21(11):e0362349. doi: 10.1371/journal.pone.0362349 (PMC13754133; doi:10.1371/journal.pone.0362349)
Supplement: Supplementary file 2 [file pone.0362349.s002.docx]

# Appendix

To verify that the main conclusions are not driven by a single parameter set, we repeated the numerical analysis under alternative parameter sets. In the robustness checks, we considered two alternative parameter sets. All alternative parameter sets satisfy the interiority, non-negativity, and logarithmic feasibility conditions specified in the note to Table 3. The robustness checks show that the qualitative conclusions remain unchanged.

Alternative Set 1 (AS 1): $A=15,\text{ }\beta=0.9,\text{ }\alpha=1.2,\text{ }c=0.8,\text{ }\Delta_{b}=2.5,\text{ }t=0.25,\text{ }s=0.15,\text{ }\eta=0.3,\text{ }\lambda=1.5$;

Alternative Set 2 (AS 2): $A=10,\text{ }\beta=0.6,\text{ }\alpha=0.9,\text{ }c=0.4,\text{ }\Delta_{b}=1.5,\text{ }t=0.15,\text{ }s=0.05,\text{ }\eta=0.1,\text{ }\lambda=1.0$.

I. AS 1	II. AS 2

Fig. A Robustness test of Fig. 1-a

I. AS 1	II. AS 2

Fig. B Robustness test of Fig. 1-b

I. AS 1	II. AS 2

Fig. C Robustness test of Fig. 2-a

I. AS 1	II. AS 2

Fig. D Robustness test of Fig. 2-b

I. AS 1	II. AS 2

Fig. E Robustness test of Fig. 2-c

I. AS 1	II. AS 2

Fig. F Robustness test of Fig. 2-d

I. AS 1	II. AS 2

Fig. G Robustness test of Fig. 3-a

I. AS 1	II. AS 2

Fig. H Robustness test of Fig. 3-b

I. AS 1	II. AS 2

Fig. I Robustness test of Fig. 3-c

I. AS 1	II. AS 2

Fig. J Robustness test of Fig. 4-a

I. AS 1	II. AS 2

Fig. K Robustness test of Fig. 4-b

I. AS 1	II. AS 2

Fig. L Robustness test of Fig. 4-c

I. AS 1	II. AS 2

Fig. M Robustness test of Fig. 4-d

I. AS 1	II. AS 2

Fig. N Robustness test of Fig. 5-a

I. AS 1	II. AS 2

Fig. O Robustness test of Fig. 5-b

I. AS 1	II. AS 2

Fig. P Robustness test of Fig. 5-c

I. AS 1	II. AS 2

Fig. Q Robustness test of Fig. 6-a

 I. AS 1	II. AS 2

Fig. R Robustness test of Fig. 6-b

I. AS 1	II. AS 2

Fig. S Robustness test of Fig. 7-a

I. AS 1	II. AS 2

Fig. T Robustness test of Fig. 7-b

I. AS 1	II. AS 2

Fig. U Robustness test of Fig. 7-c
