# Supplementary material for: Cooperative R&D contracts for power batteries under R&D outcome uncertainty and subsidies
Source: PLoS One. 2026 Nov 11;21(11):e0362349. doi: 10.1371/journal.pone.0362349 (PMC13754133; doi:10.1371/journal.pone.0362349)
Supplement: Supplementary file 3 [file pone.0362349.s003.docx]

**Supplementary material (S1 Code)--MATLAB**

%%%%--Fig.1-a-------------------------------------------------------------------

A = 10;

beta = 0.6;

Delta_b = 1;

c = 0.4;

alpha = 0.9;

s=0.05;

t = 0.1;

eta = 0.1;

lambda = linspace(0.3, 2, 100);

I_CD = Delta_b ./ lambda .* log((lambda .* (A + beta * Delta_b - alpha * c)).^2 ./ (4 * alpha * (1 - eta) * Delta_b));

I_WP = Delta_b ./ lambda .* log((lambda .* (A + beta * Delta_b - alpha * c)).^2 ./ (8 * alpha * (1 - eta) * Delta_b));

I_CS = Delta_b ./ lambda .* log((lambda .* (A + beta * Delta_b - alpha * c)).^2 ./ (8 * alpha * (1 - t) * (1 - eta) * Delta_b));

I_RS = Delta_b ./ lambda .* log((lambda .* (A + beta * Delta_b - alpha * c)).^2 ./ (4 * alpha * (2 - s)* (1 - eta) * Delta_b));

figure;

plot(lambda, I_CD, 'Color', [172/255,83/255,38/255], 'LineStyle', '-', 'LineWidth', 2); hold on;

plot(lambda, I_WP, 'Color', [167/255,32/255,97/255], 'LineStyle', ':','LineWidth', 2);

plot(lambda, I_CS, 'Color', [10/255,109/255,175/255], 'LineStyle', '--','LineWidth', 2);

plot(lambda, I_RS, 'Color', [5/255,146/255,68/255], 'LineStyle', '-.', 'LineWidth', 2);

hold off;

legend('I^{CD^*}', 'I^{DD^*}', 'I^{CS^*}', 'I^{RS^*}','Location', 'best');

xlabel('\lambda');

ylabel('I');

grid on;

%%%%--Fig.1-b-------------------------------------------------------------------

A = 10;

beta = 0.6;

delta_b = 1.5;

c = 0.4;

alpha = 0.9;

s=0.05;

eta = 0.1;

lambda = linspace(0.08, 2, 50);

t = linspace(0.2, 0.8, 50);

[LAMBDA, T] = meshgrid(lambda, t);

common_exp = (A + beta*delta_b - alpha*c)^2;

log_arg1 = (LAMBDA .* common_exp) ./ (4 * alpha * (1 - eta) * delta_b);

I1 = (delta_b ./ LAMBDA) .* log(log_arg1);

log_arg2 = (LAMBDA .* common_exp) ./ (8 * alpha * (1 - eta) * delta_b);

I2 = (delta_b ./ LAMBDA) .* log(log_arg2);

log_arg3 = (LAMBDA .* common_exp) ./ (8 * alpha * (1 - T) .* (1 - eta) * delta_b);

I3 = (delta_b ./ LAMBDA) .* log(log_arg3);

log_arg4 = (LAMBDA .* common_exp) ./ (4 * alpha * (2-s) * (1 - eta) * delta_b);

I4 = (delta_b ./ LAMBDA) .* log(log_arg4);

color_CD = [172/255,83/255,38/255];

color_WP = [167/255,32/255,97/255];

color_CS = [10/255,109/255,175/255];

color_RS = [5/255,146/255,68/255];

figure;

surf(LAMBDA, T, I1, 'FaceColor', color_CD, 'FaceAlpha',0.8, 'EdgeColor', 'none');

hold on;

surf(LAMBDA, T, I2, 'FaceColor', color_WP, 'FaceAlpha', 0.8, 'EdgeColor', 'none');

surf(LAMBDA, T, I3, 'FaceColor', color_CS, 'FaceAlpha',0.9, 'EdgeColor', 'none');

surf(LAMBDA, T, I4, 'FaceColor', color_RS, 'FaceAlpha',1, 'EdgeColor', 'none');

xlabel('\lambda');

ylabel('t');

zlabel('I');

legend('I^{CD*}', 'I^{DD*}', 'I^{CS*}','I^{RS*}', 'Location', 'best');

grid on;

%colorbar;

view(-30, 30);

hold off;

%%%%--Fig.2-a-------------------------------------------------------------------

A = 10;

beta = 0.6;

Delta_b = 1.5;

c = 0.4;

alpha = 0.9;

s=0.05;

eta = 0.1;

lambda = linspace(0.5, 2, 100);

pi_1_WP = (A + beta * Delta_b - alpha * c)^2 ./ (16 * alpha) - ((1 - eta) * Delta_b) ./ (2 * lambda);

pi_1_RS =((1-s) * (A + beta * Delta_b - alpha * c)^2 )./ (4 * alpha.*(2-s)^2) - (1 - eta) *(1 - s) * Delta_b ./ (lambda .* (2-s));

figure;

plot(lambda, pi_1_WP, 'Color', [167/255,32/255,97/255], 'LineStyle', ':', 'LineWidth', 2);hold on;

%plot(lambda, pi_1_CS, 'Color', [10/255,109/255,175/255], 'LineStyle', '--', 'LineWidth', 2);

plot(lambda, pi_1_RS, 'Color', [5/255,146/255,68/255], 'LineStyle', '-.', 'LineWidth', 2);

hold off;

legend('\pi_1^{DD^*}', '\pi_1^{RS^*}', 'Location', 'best');

xlabel('\lambda');

ylabel('\pi');

grid on;

%%%%--Fig.2-b-------------------------------------------------------------------

A = 15;

beta = 0.9;

Delta_b = 2.5;

c = 0.8;

alpha = 1.2;

s=0.15;

t = 0.25;

eta = 0.15;

lambda = linspace(0.01, 2, 100);

pi_1_CS = (A + beta * Delta_b - alpha * c)^2 ./ (16 * alpha) - (Delta_b * (1 - t) * (1 - eta)) ./ (2 * lambda) - t * (1 - eta) * Delta_b ./ lambda .* log((lambda * (A + beta * Delta_b - alpha * c)^2) ./ (Delta_b * (1 - t) * (1 - eta) * 8 * alpha));

figure;

plot(lambda, pi_1_CS, 'Color', [10/255,109/255,175/255], 'LineStyle', '--', 'LineWidth', 2);

hold off;

legend('\pi_1^{CS^*}', 'Location', 'best');

xlabel('\lambda');

ylabel('\pi');

grid on;

%%%%--Fig.2-c-------------------------------------------------------------------

A = 10;

beta = 0.6;

Delta_b = 1.5;

c = 0.4;

alpha = 0.9;

s=0.05;

t = 0.15;

eta = 0.1;

lambda = linspace(0.5, 2, 100);

pi_2_WP = (A + beta * Delta_b - alpha * c)^2 ./ (8 * alpha) - ((1 - eta) * Delta_b) ./ lambda - (1 - eta) * Delta_b ./ lambda .* log(lambda * (A + beta * Delta_b - alpha * c)^2 ./ (8 * alpha * (1 - eta) * Delta_b));

pi_2_CS = (A + beta * Delta_b - alpha * c)^2 ./ (8 * alpha) - (Delta_b * (1 - t) * (1 - eta)) ./ lambda - (1 - t) * (1 - eta) * Delta_b ./ lambda .* log(lambda * (A + beta * Delta_b - alpha * c)^2 ./ (Delta_b * (1 - t) * (1 - eta) * 8 * alpha));

pi_2_RS = (A + beta * Delta_b - alpha * c)^2 ./ (4 * alpha*(2-s)) - ((1 - eta) * Delta_b) ./ lambda - (1 - eta) * Delta_b ./ lambda .* log(lambda * (A + beta * Delta_b - alpha * c)^2 ./ (4 * alpha * (2-s)*(1 - eta) * Delta_b));

figure;

plot(lambda, pi_2_WP, 'Color', [167/255,32/255,97/255], 'LineStyle', ':', 'LineWidth', 2);hold on;

plot(lambda, pi_2_CS, 'Color', [10/255,109/255,175/255], 'LineStyle', '--', 'LineWidth', 2);

plot(lambda, pi_2_RS, 'Color', [5/255,146/255,68/255], 'LineStyle', '-.', 'LineWidth', 2);

hold off;

legend('\pi_2^{DD^*}', '\pi_2^{CS^*}', '\pi_2^{RS^*}','Location', 'best');

xlabel('\lambda');

ylabel('\pi');

grid on;

%%%%--Fig.2-d-------------------------------------------------------------------

A = 10;

beta = 0.6;

Delta_b = 1.5;

c = 0.4;

alpha = 0.9;

s=0.05;

t = 0.15;

eta = 0.1;

lambda = linspace(0.5, 2, 100);

pi_T_CD =	(A + beta * Delta_b - alpha * c)^2 ./ (4 * alpha) - ((1 - eta) * Delta_b) ./ lambda - ((1 - eta) * Delta_b) ./ lambda .* log(lambda * (A + beta * Delta_b - alpha * c)^2 ./ (4 * alpha * (1 - eta) * Delta_b));

pi_T_WP = (3 * (A + beta * Delta_b - alpha * c)^2) ./ (16 * alpha) - (3 * (1 - eta) * Delta_b) ./ (2 * lambda) - ((1 - eta) * Delta_b) ./ lambda .* log(lambda * (A + beta * Delta_b - alpha * c)^2 ./ (8 * alpha * (1 - eta) * Delta_b));

pi_T_CS = (3 * (A + beta * Delta_b - alpha * c)^2) ./ (16 * alpha) - (3 * (1 - t) * (1 - eta) * Delta_b) ./ (2 * lambda) - ((1 - eta) * Delta_b) ./ lambda .* log(lambda * (A + beta * Delta_b - alpha * c)^2 ./ (8 * alpha * (1 - t) * (1 - eta) * Delta_b));

pi_T_RS = ((3-2*s) * (A + beta * Delta_b - alpha * c)^2) ./ (4 * alpha.*(2-s)^2) - ((3-2*s) * (1 - t) * (1 - eta) * Delta_b) ./ ((2-s) * lambda) - ((1 - eta) * Delta_b) ./ lambda .* log(lambda * (A + beta * Delta_b - alpha * c)^2 ./ (4 * alpha * (2 - s) * (1 - eta) * Delta_b));

figure;

plot(lambda, pi_T_CD, 'Color', [172/255,83/255,38/255], 'LineStyle', '-', 'LineWidth', 2); hold on;

plot(lambda, pi_T_WP, 'Color', [167/255,32/255,97/255], 'LineStyle', ':', 'LineWidth', 2);

plot(lambda, pi_T_CS, 'Color', [10/255,109/255,175/255], 'LineStyle', '--', 'LineWidth', 2);

plot(lambda, pi_T_RS, 'Color', [5/255,146/255,68/255], 'LineStyle', '-.', 'LineWidth', 2);

hold off;

legend('\pi_T^{CD^*}', '\pi_T^{DD^*}', '\pi_T^{CS^*}', '\pi_T^{RS^*}','Location', 'best');

xlabel('\lambda');

ylabel('\pi');

grid on;

%%%%--Fig.3-a-------------------------------------------------------------------

A = 10;

beta = 0.6;

Delta_b = 1.5;

c = 0.4;

alpha = 0.9;

s=0.05;

t = 0.15;

lambda =1.0;

eta = linspace(0.1, 0.9, 100);

I_CD = Delta_b ./ lambda .* log((lambda * (A + beta * Delta_b - alpha * c)).^2 ./ (4 * alpha * (1 - eta) * Delta_b));

I_WP = Delta_b ./ lambda .* log((lambda * (A + beta * Delta_b - alpha * c)).^2 ./ (8 * alpha * (1 - eta) * Delta_b));

I_CS = Delta_b ./ lambda .* log((lambda * (A + beta * Delta_b - alpha * c)).^2 ./ (Delta_b * (1 - t) * (1 - eta) * 8 * alpha));

I_RS = Delta_b ./ lambda .* log((lambda * (A + beta * Delta_b - alpha * c)).^2 ./ (4 * alpha * (2-s)*(1 - eta) * Delta_b));

figure;

plot(eta, I_CD, 'Color', [172/255,83/255,38/255], 'LineStyle', '-', 'LineWidth', 2); hold on;

plot(eta, I_WP, 'Color', [167/255,32/255,97/255], 'LineStyle', ':', 'LineWidth', 2);

plot(eta, I_CS, 'Color', [10/255,109/255,175/255], 'LineStyle', '--', 'LineWidth', 2);

plot(eta, I_RS, 'Color', [5/255,146/255,68/255], 'LineStyle', '-.', 'LineWidth', 2);

hold off;

legend('I^{CD^*}', 'I^{DD^*}', 'I^{CS^*}','I^{RS^*}','Location', 'best');

xlabel('\eta');

ylabel('I');

grid on;

%%%%--Fig.3-b-------------------------------------------------------------------

A = 10;

beta = 0.6;

delta_b = 1.5;

c = 0.4;

alpha = 0.9;

s=0.05;

lambda =1.0;

eta = linspace(0.1, 0.9, 100);

t = linspace(0.1, 0.9, 100);

[ETA, T] = meshgrid(eta, t);

common_part = (A + beta*delta_b - alpha*c)^2;

log_arg1 = (lambda * common_part) ./ (4 * alpha * (1 - ETA) * delta_b);

I1 = (delta_b / lambda) .* log(log_arg1);

log_arg2 = (lambda * common_part) ./ (8 * alpha * (1 - ETA) * delta_b);

I2 = (delta_b / lambda) .* log(log_arg2);

log_arg3 = (lambda * common_part) ./ (8 * alpha * (1 - T) .* (1 - ETA) * delta_b);

I3 = (delta_b / lambda) .* log(log_arg3);

log_arg4 = (lambda * common_part) ./ (4 * alpha * (2-s) * (1 - ETA) * delta_b);

I4 = (delta_b / lambda) .* log(log_arg4);

color_CD = [172/255,83/255,38/255];

color_WP = [167/255,32/255,97/255];

color_CS = [10/255,109/255,175/255];

color_RS = [5/255,146/255,68/255];

figure;

surf(ETA, T, I1, 'FaceColor', color_CD, 'FaceAlpha', 0.8, 'EdgeColor', 'none');

hold on;

surf(ETA, T, I2, 'FaceColor', color_WP, 'FaceAlpha', 0.8, 'EdgeColor', 'none');

surf(ETA, T, I3, 'FaceColor', color_CS, 'FaceAlpha',0.9, 'EdgeColor', 'none');

surf(ETA, T, I4, 'FaceColor', color_RS, 'FaceAlpha',1, 'EdgeColor', 'none');

xlabel('\eta');

ylabel('t');

zlabel('I');

legend('I^{CD*}', 'I^{DD*}', 'I^{CS*}', 'I^{RS*}','Location', 'best');

grid on;

%colorbar;

view(-30, 30);

hold off;

%%%%--Fig.3-c-------------------------------------------------------------------

A = 10;

beta = 0.6;

delta_b = 1.5;

alpha = 0.9;

c = 0.4;

eta = 0.1;

lambda = 1.0;

[s, t] = meshgrid(linspace(0.01, 0.9, 100), linspace(0.01, 0.9, 100));

K = A + beta * delta_b - alpha * c;

log_arg = lambda * K^2 ./ (4 * alpha * (1 - eta) * delta_b);

I_CS = ( delta_b ./ lambda) .* log(log_arg ./ (2*(1-t)));

I_RS = ( delta_b ./ lambda) .* log(log_arg./(2-s));

color_CS = [10/255,109/255,175/255];

color_RS = [5/255,146/255,68/255];

figure;

surf(s, t, I_CS, 'FaceColor', color_CS, 'FaceAlpha', 1, 'EdgeColor', 'none');

hold on;

surf(s, t, I_RS, 'FaceColor', color_RS, 'FaceAlpha',1, 'EdgeColor', 'none');

xlabel('s');

ylabel('t');

zlabel('I');

legend('I^{CS*}', 'I^{RS*}', 'Location', 'best');

grid on;

view(3);

hold off;

%%%%--Fig.4-a-------------------------------------------------------------------

A = 10;

beta = 0.6;

Delta_b = 1.5;

c = 0.4;

alpha = 0.9;

s=0.05;

t = 0.15;

lambda =1.0;

eta = linspace (0.1, 0.9, 100);

pi_1_WP = (A + beta * Delta_b - alpha * c)^2 ./ (16 * alpha) - ((1 - eta) * Delta_b) ./ (2 * lambda);

%pi_1_CS = (A + beta * Delta_b - alpha * c)^2 ./ (16 * alpha) - (Delta_b * (1 - t) * (1 - eta)) ./ (2 * lambda) - t * (1 - eta) * Delta_b ./ lambda .* log((lambda * (A + beta * Delta_b - alpha * c)^2) ./ (Delta_b * (1 - t) * (1 - eta) * 8 * alpha));

pi_1_RS =((1-s) * (A + beta * Delta_b - alpha * c)^2 )./ (4 * alpha.*(2-s)^2) - (1 - eta) *(1 - s) * Delta_b ./ (lambda .* (2-s));

figure;

plot(eta, pi_1_WP, 'Color', [167/255,32/255,97/255], 'LineStyle', ':', 'LineWidth', 2);hold on;

%plot(eta, pi_1_CS, 'Color', [10/255,109/255,175/255],'LineStyle', '--', 'LineWidth', 2);

plot(eta, pi_1_RS, 'Color', [5/255,146/255,68/255], 'LineStyle', '-.', 'LineWidth', 2);

hold off;

legend('\pi_1^{DD^*}', '\pi_1^{RS^*}', 'Location', 'best');

xlabel('\eta');

ylabel('\pi');

grid on;

%%%%--Fig.4-b-------------------------------------------------------------------

A = 1; % A needs to be adjusted downward to display the decreasing trend

beta = 0.6;

Delta_b = 1.5;

c = 0.4;

alpha = 0.9;

s=0.15;

t = 0.4;

lambda =1.0;

eta = linspace (0.1, 1.0, 100);

pi_1_CS = (A + beta * Delta_b - alpha * c)^2 ./ (16 * alpha) - (Delta_b * (1 - t) * (1 - eta)) ./ (2 * lambda) - t * (1 - eta) * Delta_b ./ lambda .* log((lambda * (A + beta * Delta_b - alpha * c)^2) ./ (Delta_b * (1 - t) * (1 - eta) * 8 * alpha));

figure;

plot(eta, pi_1_CS, 'Color', [10/255,109/255,175/255],'LineStyle', '--', 'LineWidth', 2);

hold off;

legend('\pi_1^{CS^*}','Location', 'best');

xlabel('\eta');

ylabel('\pi');

grid on;

%%%%--Fig.4-c-------------------------------------------------------------------

A = 10;

beta = 0.6;

Delta_b = 1.5;

c = 0.4;

alpha = 0.9;

s=0.05;

t = 0.15;

lambda =1.0;

eta = linspace(0.1, 0.9, 100);

pi_2_WP = (A + beta * Delta_b - alpha * c)^2 ./ (8 * alpha) - ((1 - eta) * Delta_b) ./ lambda - (1 - eta) * Delta_b ./ lambda .* log(lambda * (A + beta * Delta_b - alpha * c)^2 ./ (8 * alpha * (1 - eta) * Delta_b));

pi_2_CS = (A + beta * Delta_b - alpha * c)^2 ./ (8 * alpha) - (Delta_b * (1 - t) * (1 - eta)) ./ lambda - (1 - t) * (1 - eta) * Delta_b ./ lambda .* log(lambda * (A + beta * Delta_b - alpha * c)^2 ./ (Delta_b * (1 - t) * (1 - eta) * 8 * alpha));

pi_2_RS = (A + beta * Delta_b - alpha * c)^2 ./ (4 * alpha*(2-s)) - ((1 - eta) * Delta_b) ./ lambda - (1 - eta) * Delta_b ./ lambda .* log(lambda * (A + beta * Delta_b - alpha * c)^2 ./ (4 * alpha * (2-s)*(1 - eta) * Delta_b));

figure;

plot(eta, pi_2_WP, 'Color', [167/255,32/255,97/255], 'LineStyle', ':','LineWidth', 2);hold on;

plot(eta, pi_2_CS, 'Color', [10/255,109/255,175/255], 'LineStyle', '--','LineWidth', 2);

plot(eta, pi_2_RS, 'Color', [5/255,146/255,68/255], 'LineStyle', '-.', 'LineWidth', 2);

hold off;

legend('\pi_2^{DD^*}', '\pi_2^{CS^*}', '\pi_2^{RS^*}','Location', 'best');

xlabel('\eta');

ylabel('\pi');

grid on;

%%%%--Fig.4-d-------------------------------------------------------------------

A = 10;

beta = 0.6;

Delta_b = 1.5;

c = 0.4;

alpha = 0.9;

s=0.05;

t = 0.15;

lambda =1.0;

eta = linspace(0.1, 0.9, 100);

pi_T_CD =	(A + beta * Delta_b - alpha * c)^2 ./ (4 * alpha) - ((1 - eta) * Delta_b) ./ lambda - ((1 - eta) * Delta_b) ./ lambda .* log(lambda * (A + beta * Delta_b - alpha * c)^2 ./ (4 * alpha * (1 - eta) * Delta_b));

pi_T_WP = (3 * (A + beta * Delta_b - alpha * c)^2) ./ (16 * alpha) - (3 * (1 - eta) * Delta_b) ./ (2 * lambda) - ((1 - eta) * Delta_b) ./ lambda .* log(lambda * (A + beta * Delta_b - alpha * c)^2 ./ (8 * alpha * (1 - eta) * Delta_b));

pi_T_CS = (3 * (A + beta * Delta_b - alpha * c)^2) ./ (16 * alpha) - (3 * (1 - t) * (1 - eta) * Delta_b) ./ (2 * lambda) - ((1 - eta) * Delta_b) ./ lambda .* log(lambda * (A + beta * Delta_b - alpha * c)^2 ./ (8 * alpha * (1 - t) * (1 - eta) * Delta_b));

pi_T_RS = ((3-2*s) * (A + beta * Delta_b - alpha * c)^2) ./ (4 * alpha.*(2-s)^2) - ((3-2*s) * (1 - t) * (1 - eta) * Delta_b) ./ ((2-s) * lambda) - ((1 - eta) * Delta_b) ./ lambda .* log(lambda * (A + beta * Delta_b - alpha * c)^2 ./ (4 * alpha * (2 - s) * (1 - eta) * Delta_b));

figure;

plot(eta, pi_T_CD, 'Color', [172/255,83/255,38/255], 'LineStyle', '-', 'LineWidth', 2); hold on;

plot(eta, pi_T_WP, 'Color', [167/255,32/255,97/255], 'LineStyle', ':','LineWidth', 2);

plot(eta, pi_T_CS, 'Color', [10/255,109/255,175/255], 'LineStyle', '--','LineWidth', 2);

plot(eta, pi_T_RS, 'Color', [5/255,146/255,68/255], 'LineStyle', '-.', 'LineWidth', 2);

hold off;

legend('\pi_T^{CD^*}', '\pi_T^{DD^*}', '\pi_T^{CS^*}', '\pi_T^{RS^*}','Location', 'best');

xlabel('\eta');

ylabel('\pi');

grid on;

%%%%--Fig.5-a-------------------------------------------------------------------

A = 10;

beta = 0.6;

delta_b = 1.5;

c = 0.4;

alpha = 0.9;

t = 0.15;

lambda = linspace(0.06, 1, 100);

eta = linspace(0.1, 0.9, 100);

[LAMBDA, ETA] = meshgrid(lambda, eta);

common_term = (A + beta*delta_b - alpha*c)^2;

pi_WP = common_term/(16*alpha) - ((1 - ETA).*delta_b)./(2*LAMBDA);

term1_CS = common_term/(16*alpha);

term2_CS = ((1 - t).*(1 - ETA).*delta_b)./(2*LAMBDA);

log_arg_CS = (LAMBDA .* common_term)./(8*alpha.*(1 - t).*(1 - ETA).*delta_b);

log_term_CS = (t.*(1 - ETA).*delta_b)./LAMBDA .* log(log_arg_CS);

pi_CS = term1_CS - term2_CS - log_term_CS;

color_WP = [167/255,32/255,97/255];

color_CS = [10/255,109/255,175/255];

figure;

surf(LAMBDA, ETA, pi_WP, 'FaceColor', color_WP, 'FaceAlpha', 0.8, 'EdgeColor', 'none');

hold on;

surf(LAMBDA, ETA, pi_CS, 'FaceColor', color_CS, 'FaceAlpha',0.8, 'EdgeColor', 'none');

xlabel('\lambda');

ylabel('\eta');

zlabel('I');

legend('\pi_1^{DD*}', '\pi_1^{CS*}', 'Location', 'best', 'FontSize', 12);

grid on;

%colorbar;

view(-50, 50);

hold off;

%%%%--Fig.5-b-------------------------------------------------------------------

A = 10;

beta = 0.6;

delta_b = 1.5;

c = 0.4;

alpha = 0.9;

s=0.05;

t = 0.15;

lambda = linspace(0.06, 1, 100);

eta = linspace(0.1, 0.9, 100);

[LAMBDA, ETA] = meshgrid(lambda, eta);

common_term = (A + beta*delta_b - alpha*c)^2;

pi_WP = common_term/(16*alpha) - ((1 - ETA).*delta_b)./(2*LAMBDA);

term1_RS = (1-s)*common_term/(4*alpha*(2-s)*(2-s));

term2_RS = ((1 - s).*(1 - ETA).*delta_b)./((2-s)*LAMBDA);

pi_RS = term1_RS - term2_RS;

color_WP = [167/255,32/255,97/255];

color_RS = [5/255,146/255,68/255];

figure;

surf(LAMBDA, ETA, pi_WP, 'FaceColor', color_WP, 'FaceAlpha', 0.8, 'EdgeColor', 'none');

hold on;

surf(LAMBDA, ETA, pi_RS, 'FaceColor', color_RS, 'FaceAlpha',0.8, 'EdgeColor', 'none');

xlabel('\lambda');

ylabel('\eta');

zlabel('I');

legend('\pi_1^{DD*}', '\pi_1^{RS*}', 'Location', 'best', 'FontSize', 12);

grid on;

%colorbar;

view(-50, 50);

hold off;

%%%%--Fig.5-c-------------------------------------------------------------------

A = 10;

beta = 0.8;

delta_b = 1.5;

alpha = 0.9;

c = 0.4;

eta = 0.1;

lambda = 1.0;

[s, t] = meshgrid(linspace(0.01, 0.99, 50), linspace(0.01, 0.99, 50));

K = A + beta * delta_b - alpha * c;

log_arg = lambda * K^2 ./ (8 * alpha * (1 - t) * (1 - eta) * delta_b);

pi_1_CS = (K^2) / (16 * alpha) ...

- ((1 - t) .* (1 - eta) * delta_b) / (2 * lambda) ...

- (t .* (1 - eta) * delta_b / lambda) .* log(log_arg);

pi_1_RS = ((1 - s) * K^2) ./ (4 * alpha * (2 - s).^2) ...

- (delta_b * (1 - s) * (1 - eta)) ./ (lambda * (2 - s));

color_CS = [10/255,109/255,175/255];

color_RS = [5/255,146/255,68/255];

figure;

surf(s, t, pi_1_CS, 'FaceColor', color_CS, 'FaceAlpha', 1, 'EdgeColor', 'none');

hold on;

surf(s, t, pi_1_RS, 'FaceColor', color_RS, 'FaceAlpha',1, 'EdgeColor', 'none');

xlabel('s');

ylabel('t');

zlabel('\pi');

legend('\pi_1^{CS*}', '\pi_1^{RS*}', 'Location', 'best');

grid on;

view(3);

hold off;

%%%%--Fig.6-a-------------------------------------------------------------------

A = 10;

beta = 0.6;

delta_b = 1.5;

c = 0.4;

alpha = 0.92;

s=0.05;

t = 0.15;

lambda = linspace(0.5, 2, 50);

eta = linspace(0.1, 0.9, 50);

[LAMBDA, ETA] = meshgrid(lambda, eta);

common_term = (A + beta*delta_b - alpha*c)^2;

term1_WP = common_term / (8*alpha);

term2_WP = ((1 - ETA) .* delta_b) ./ LAMBDA;

log_arg_WP = (LAMBDA .* common_term) ./ (8*alpha.*(1 - ETA).*delta_b);

pi_WP = term1_WP - term2_WP - term2_WP .* log(log_arg_WP);

term1_CS = common_term / (8*alpha);

term2_CS = ((1 - t) .* (1 - ETA) .* delta_b) ./ LAMBDA;

log_arg_CS = (LAMBDA .* common_term) ./ (8*alpha.*(1 - t).*(1 - ETA).*delta_b);

pi_CS = term1_CS - term2_CS - term2_CS .* log(log_arg_CS);

term1_RS = common_term / (4*alpha*(2-s));

term2_RS = ((1 - ETA) .* delta_b) ./ LAMBDA;

log_arg_RS = (LAMBDA .* common_term) ./ (4*alpha.*(2 - s).*(1 - ETA).*delta_b);

pi_RS = term1_RS - term2_RS - term2_RS .* log(log_arg_RS);

color_WP = [167/255,32/255,97/255];

color_CS = [10/255,109/255,175/255];

color_RS = [5/255,146/255,68/255];

figure;

surf(LAMBDA, ETA, pi_WP, 'FaceColor', color_WP, 'FaceAlpha', 0.8, 'EdgeColor', 'none');

hold on;

surf(LAMBDA, ETA, pi_CS, 'FaceColor', color_CS, 'FaceAlpha',0.8, 'EdgeColor', 'none');

surf(LAMBDA, ETA, pi_RS, 'FaceColor', color_RS, 'FaceAlpha',0.8, 'EdgeColor', 'none');

xlabel('\lambda');

ylabel('\eta');

zlabel('I');

legend('\pi_2^{DD*}', '\pi_2^{CS*}', '\pi_2^{RS*}','Location', 'best', 'FontSize', 12);

grid on;

view(-50, 50);

hold off;

%%%%--Fig.6-b-------------------------------------------------------------------

A = 10;

beta = 0.6;

delta_b = 1.5;

alpha = 0.9;

c = 0.4;

eta = 0.1;

lambda = 1.0;

[s, t] = meshgrid(linspace(0.01, 0.99, 50), linspace(0.01, 0.99, 50));

K = A + beta * delta_b - alpha * c;

log_arg = lambda * K^2 ./ (8 * alpha * (1 - eta) * delta_b);

pi_2_CS = (K^2) / (8 * alpha) ...

- ((1 - t) .* (1 - eta) * delta_b) / (lambda) ...

- ((1-t) .* (1 - eta) * delta_b / lambda) .* log(log_arg ./(1-t));

pi_2_RS = ( K^2) ./ (4 * alpha * (2 - s)) ...

- (delta_b * (1 - eta)) ./ (lambda )...

- ((1 - eta) * delta_b / lambda) .* log(log_arg ./(2-s));

color_CS = [10/255,109/255,175/255];

color_RS = [5/255,146/255,68/255];

figure;

surf(s, t, pi_2_CS, 'FaceColor', color_CS, 'FaceAlpha', 1, 'EdgeColor', 'none');

hold on;

surf(s, t, pi_2_RS, 'FaceColor', color_RS, 'FaceAlpha',1, 'EdgeColor', 'none');

xlabel('s');

ylabel('t');

zlabel('\pi');

legend('\pi_2^{CS*}', '\pi_2^{RS*}', 'Location', 'best');

grid on;

view(3);

hold off;

%%%%--Fig.7-a(t<0.5)-------------------------------------------------------------------

A = 10;

beta = 0.6;

delta_b = 1.5;

c = 0.4;

alpha = 0.9;

s=0.05;

t = 0.15;

lambda = linspace(0.2, 1, 50);

eta = linspace(0.2, 0.9, 50);

[LAMBDA, ETA] = meshgrid(lambda, eta);

common_term = (A + beta*delta_b - alpha*c)^2;

term1_CD = common_term / (4*alpha);

term2_CD = ((1 - ETA).*delta_b) ./ LAMBDA;

log_arg_CD = (LAMBDA .* common_term) ./ (4*alpha.*(1 - ETA).*delta_b);

pi_CD = term1_CD - term2_CD - term2_CD .* log(log_arg_CD);

term1_WP = 3*common_term / (16*alpha);

term2_WP = (3*(1 - ETA).*delta_b) ./ (2*LAMBDA);

log_arg_WP = (LAMBDA .* common_term) ./ (8*alpha.*(1 - ETA).*delta_b);

pi_WP = term1_WP - term2_WP - term2_CD .* log(log_arg_WP);

term1_CS = 3*common_term / (16*alpha);

term2_CS = (3*(1 - t).*(1 - ETA).*delta_b) ./ (2*LAMBDA);

log_arg_CS = (LAMBDA .* common_term) ./ (8*alpha.*(1 - t).*(1 - ETA).*delta_b);

pi_CS = term1_CS - term2_CS - term2_CD .* log(log_arg_CS);

term1_RS = (3-2*s)*common_term / (4*alpha*(2-s)*(2-s));

term2_RS = ((3 - 2*s).*(1 - ETA).*delta_b) ./ ((2-s)*LAMBDA);

log_arg_RS = (LAMBDA .* common_term) ./ (4*alpha.*(2 - s).*(1 - ETA).*delta_b);

pi_RS = term1_RS - term2_RS - term2_CD .* log(log_arg_RS);

color_CD = [172/255,83/255,38/255];

color_WP = [167/255,32/255,97/255];

color_CS = [10/255,109/255,175/255];

color_RS = [5/255,146/255,68/255];

figure;

surf(LAMBDA, ETA, pi_CD, 'FaceColor', color_CD, 'FaceAlpha', 0.8, 'EdgeColor', 'none');

hold on;

surf(LAMBDA, ETA, pi_WP, 'FaceColor', color_WP, 'FaceAlpha', 1, 'EdgeColor', 'none');

surf(LAMBDA, ETA, pi_CS, 'FaceColor', color_CS, 'FaceAlpha',0.8, 'EdgeColor', 'none');

surf(LAMBDA, ETA, pi_RS, 'FaceColor', color_RS, 'FaceAlpha',0.8, 'EdgeColor', 'none');

xlabel('\lambda');

ylabel('\eta');

zlabel('\pi');

legend('\pi_T^{CD*}', '\pi_T^{DD*}', '\pi_T^{CS*}', '\pi_T^{RS*}','Location', 'best', 'FontSize', 12);

grid on;

%colorbar;

view(-50, 50);

hold off;

%%%%--Fig.7-b(t>0.5)-------------------------------------------------------------------

A = 10;

beta = 0.6;

delta_b = 1.5;

c = 0.6;

alpha = 0.9;

s=0.05;

t = 0.7;

lambda = linspace(0.2, 1, 50);

eta = linspace(0.2, 0.9, 50);

[LAMBDA, ETA] = meshgrid(lambda, eta);

common_term = (A + beta*delta_b - alpha*c)^2;

term1_CD = common_term / (4*alpha);

term2_CD = ((1 - ETA).*delta_b) ./ LAMBDA;

log_arg_CD = (LAMBDA .* common_term) ./ (4*alpha.*(1 - ETA).*delta_b);

pi_CD = term1_CD - term2_CD - term2_CD .* log(log_arg_CD);

term1_WP = 3*common_term / (16*alpha);

term2_WP = (3*(1 - ETA).*delta_b) ./ (2*LAMBDA);

log_arg_WP = (LAMBDA .* common_term) ./ (8*alpha.*(1 - ETA).*delta_b);

pi_WP = term1_WP - term2_WP - term2_CD .* log(log_arg_WP);

term1_CS = 3*common_term / (16*alpha);

term2_CS = (3*(1 - t).*(1 - ETA).*delta_b) ./ (2*LAMBDA);

log_arg_CS = (LAMBDA .* common_term) ./ (8*alpha.*(1 - t).*(1 - ETA).*delta_b);

pi_CS = term1_CS - term2_CS - term2_CD .* log(log_arg_CS);

term1_RS = (3-2*s)*common_term / (4*alpha*(2-s)*(2-s));

term2_RS = ((3 - 2*s).*(1 - ETA).*delta_b) ./ ((2-s)*LAMBDA);

log_arg_RS = (LAMBDA .* common_term) ./ (4*alpha.*(2 - s).*(1 - ETA).*delta_b);

pi_RS = term1_RS - term2_RS - term2_CD .* log(log_arg_RS);

color_CD = [172/255,83/255,38/255];

color_WP = [167/255,32/255,97/255];

color_CS = [10/255,109/255,175/255];

color_RS = [5/255,146/255,68/255];

figure;

surf(LAMBDA, ETA, pi_CD, 'FaceColor', color_CD, 'FaceAlpha', 0.8, 'EdgeColor', 'none');

hold on;

surf(LAMBDA, ETA, pi_WP, 'FaceColor', color_WP, 'FaceAlpha', 1, 'EdgeColor', 'none');

surf(LAMBDA, ETA, pi_CS, 'FaceColor', color_CS, 'FaceAlpha',0.8, 'EdgeColor', 'none');

surf(LAMBDA, ETA, pi_RS, 'FaceColor', color_RS, 'FaceAlpha',0.8, 'EdgeColor', 'none');

xlabel('\lambda');

ylabel('\eta');

zlabel('\pi');

legend('\pi_T^{CD*}', '\pi_T^{DD*}', '\pi_T^{CS*}', '\pi_T^{RS*}','Location', 'best', 'FontSize', 12);

grid on;

%colorbar;

view(-50, 50);

hold off;

%%%%--Fig.7-c-------------------------------------------------------------------

A = 10;

beta = 0.6;

delta_b = 1.5;

alpha = 0.9;

c = 0.4;

eta = 0.1;

lambda = 1.0;

[s, t] = meshgrid(linspace(0.00001, 0.05, 100), linspace(0.00001, 0.05, 100));

K = A + beta * delta_b - alpha * c;

log_arg = lambda * K^2 ./ (8 * alpha * (1 - eta) * delta_b);

pi_T_CS = (3 * K^2) / (16 * alpha) ...

- (3* (1 - t) .* (1 - eta) * delta_b) / (2*lambda) ...

- ((1 - eta) * delta_b / lambda) .* log(log_arg ./ (1-t));

pi_T_RS = ( (3-2*s) * K^2) ./ (4 * alpha * (2 - s).^2) ...

- ((3-2*s)*delta_b * (1 - eta)) ./ (lambda*(2-s) )...

- ((1 - eta) * delta_b / lambda) .* log(2*log_arg./(2-s));

color_CS = [10/255,109/255,175/255];

color_RS = [5/255,146/255,68/255];

figure;

surf(s, t, pi_T_CS, 'FaceColor', color_CS, 'FaceAlpha', 1, 'EdgeColor', 'none');

hold on;

surf(s, t, pi_T_RS, 'FaceColor', color_RS, 'FaceAlpha',1, 'EdgeColor', 'none');

xlabel('s');

ylabel('t');

zlabel('\pi');

legend('\pi_2^{CS*}', '\pi_2^{RS*}', 'Location', 'best');

grid on;

view(3);

hold off;
